# Comparative Analyses and Phylogenetic Dependence in Traits and Trends of the Dipterocarpaceae

**DOI:** 10.1002/ece3.70784

**Published:** 2025-01-09

**Authors:** Nazrin Malik, David Edwards, Robert P. Freckleton

**Affiliations:** ^1^ Ecology and Evolutionary Biology, School of Biosciences University of Sheffield Sheffield UK; ^2^ Department of Forestry Science & Biodiversity, Faculty of Forestry Universiti Putra Malaysia Serdang Selangor Malaysia; ^3^ Department of Plant Sciences and Centre for Global Wood Security University of Cambridge Cambridge UK; ^4^ Conservation Research Institute University of Cambridge Cambridge UK

**Keywords:** comparative analysis, Dipterocarpaceae, phylogenetic niche conservatism, plant traits, species diversity

## Abstract

The role of trait evolution in shaping the functional and ecological diversity of tropical forests remains poorly understood. Analyses of trait variation as a function of evolutionary history and environmental variables should reveal the drivers of species distributions, as well as generate insights valuable to conservation. Here, we focus on the Dipterocarpaceae, the key plant family underpinning the hyperdiversity of South‐East Asian tropical forest canopies and of major conservation concern due to over‐exploitation for timber, cultivation, and climate change. Our objectives are to (i) assess whether dipterocarp species traits are phylogenetically conserved through a phylogenetic signal, indicating phylogenetic niche conservatism (PNC); (ii) determine the drivers of dipterocarp species distribution; (iii) examine the relationship between morphological traits with habitat factors; and (iv) assess the correlation between conservation status and phylogeny. We compiled a dataset of species‐level plant traits of the Dipterocarpaceae together with population‐level ecological trends. We found substantial evidence of phylogenetic conservatism of plant traits in dipterocarp species, with a moderate to strong phylogenetic signal, and that the elevational gradient shapes dipterocarp species distribution pan‐tropically. Morphological traits including height and diameter show phylogenetically dependent relationships with soil type, while shade tolerance traits are related to survival. We find that conservation status is related to phylogeny and correlated with population trend status, suggesting that decreasing population trends correlated with conservation status. Overall, our analyses show that functional traits and ecological trends of dipterocarp species are shaped by the phylogenetic history. Our study highlights that conservation strategies require consideration of the consequences of these relationships for long‐term population changes.

## 1 Introduction

Tropical forests are the most mega‐diverse terrestrial ecosystems globally (Poore 1991; Slik et al. 2015). Coexistence of many species within the same community has led to the vast floristic richness in tropical forests (Whitmore 1984; Poore 1991), with much work undertaken by ecologists to understand and explain this variation. A great deal of this effort has been expended in trying to understand the ecological factors that drive diversity. Hypotheses such as the Janzen–Connell mechanism (Janzen 1970; Connell 1971) and the neutral theory (Hubbell 2001) offer different perspectives on the factors that drive diversity. Although there is growing support for the Janzen–Connell mechanism (Swamy and Terborgh 2010; Comita et al. 2014; Zhu et al. 2015), both theories are basically ecological in nature (Hubbell 2001). Thus, they do not consider the role of evolution or traits in shaping distributions or diversity within tropical forests.

At the pantropical scale, species adapt to contrasting environments through the evolution of their functional traits, and variation in these with environmental conditions is a fundamental feature of biological diversity (Ackerly 2004). Pavoine and Bonsall (2011) highlighted the need to assess the relationship between evolutionary processes, species traits variances, and species interaction with the environment to fully understand the factors that drive variation in traits across different environments. Thus, in addition to understanding ecological diversity, there is also a need to document and explain diversity in species traits.

A key concept in understanding large‐scale patterns in trait variation is phylogenetic niche conservatism (PNC; Harvey and Pagel 1991). As defined by Wiens and Graham (2005), this is the tendency of closely related species with common evolutionary history to share similar ecological, morphological, physiological, and life‐history traits. There are multiple mechanisms and drivers of PNC, but essentially, it results from physiological and ecological constraints on species that limit them to a restricted set of ecological or environmental niches (Harvey and Pagel 1991; Wiens and Graham 2005; Cooper, Freckleton, and Jetz 2011). Consequently, whole taxa can be limited to a similar subset of environments (Harvey and Pagel 1991; Wiens and Graham 2005; Cooper, Freckleton, and Jetz 2011). In the face of ongoing Anthropogenic threats to biodiversity, this means that extinction is likely to be non‐randomly distributed with respect to phylogeny and thus it is important to characterize PNC.

A suite of tests for PNC exist, which revolve around measuring a phylogenetic signal in key traits (Blomberg, Garland, and Ives 2003; Cooper, Freckleton, and Jetz 2011; Pavoine and Bonsall 2011). These tests are based on phylogenetic comparative methods (PCMs), which have been developed to measure a phylogenetic signal in trait variance (Harvey and Pagel 1991; Freckleton, Harvey, and Pagel 2002; Blomberg, Garland, and Ives 2003; Fritz and Purvis 2010; Cooper, Thomas, and FitzJohn 2016). Such approaches measure PNC by measuring how trait variation is associated with phylogeny (Kreier and Schneider 2006; Cooper, Freckleton, and Jetz 2011; Liu et al. 2012, 2016) and can potentially address the prediction of PNC that closely related species should share similar traits than distantly related ones.

Although testing for a phylogenetic signal seems like a logical approach to investigate PNC, there are potential pitfalls and several studies have pointed that these methods can be limited and are dependent on the assumptions made, as well as the existence of possible statistical biases (Freckleton 2009; Cooper, Freckleton, and Jetz 2011; Losos 2011; Blomberg et al. 2012; Cooper, Thomas, and FitzJohn 2016). It is important to recognize at the outset that when modeling comparative data, several different processes could yield the same outcome in the phylogenetic dispersion of traits (Revell et al. 2008). In modeling PNC, it is necessary to specify the process by which it is believed PNC may evolve, as well as to clearly specify ‘null’ alternatives. This is because both phylogenetic signals, and the lack of them, could conceivably both be the consequence or not of PNC depending on the specific underlying process (Cooper, Freckleton, and Jetz 2011).

We study the Dipterocarpaceae family, which globally comprises 695 species within 16 genera. Dipterocarp species are highly regarded for their timber market value, which has been a major economic contributor to South‐East Asian countries (Appanah and Turnbull 1998). The distribution of the dipterocarps is mainly limited to tropical and sub‐tropical regions, in which mean annual rainfall exceeds 1000 mm. The three dipterocarp subfamilies occur in specific regions: Dipterocarpoideae in Asia, Pakaraimoidae in South America, and Monotoideae in Africa (Ghazoul 2016). A study by Bansal et al. (2022) highlights how past environmental conditions, including climate and land connectivity, shaped the distribution and diversification of dipterocarp species. For instance, changes in sea levels and the configuration of landmasses over geological time scales played a crucial role in this process.

There is evidence of environmental constraints on dipterocarp distributions. A large number of species occur below 1000 m altitude. For instance, high dipterocarp species richness is observed in lowland rainforests with elevation up to 300 m in Peninsular Malaysia, Thailand, Sumatra, and Borneo (Ashton 1982; Ashton, Givnish, and Appanah 1988; Ghazoul 2016). Soil type appears to have contributed to this distribution pattern: the richest dipterocarp communities occur on the yellow sandy humult soil regions compared to homogenous clay soil regions (Russo et al. 2005; Katabuchi et al. 2012; Ghazoul 2016). An important question is whether niche conservatism operates in limiting dipterocarp species adaptations to these environmental factors, and whether any such evolutionary conservation might limit species distributions.

Based on testing for the existence and strength of PNC, we investigated how plant traits vary among dipterocarps. Our objectives were to (1) measure the phylogenetic signal in the plant traits of all known dipterocarp species to assess the degree to which PNC shapes trait distributions; (2) analyze how different ecological adaptations were associated with species distribution; (3) assess to which extent the morphological traits and species performance correlated with habitat and soil type to understand how traits are shaped by environmental factors; and (4) analyze the correlation between conservation status and phylogeny in the Dipterocarpaceae family to determine whether PNC contributes to extinction threats.

## 2 Materials and Methods

### 2.1 Study Group

We based our study on the Dipterocarpaceae. These are sub‐canopy, canopy, or emergent trees, with many species exceeding 50 m in height (Ashton, Givnish, and Appanah 1988; Ghazoul 2016). Their distribution encompasses tropical and sub‐tropical countries, where the mean annual rainfall generally exceeds 1000 mm, including Papua New Guinea, the South‐East Asian countries, China, India, Sri Lanka, Seychelles, Madagascar, Angola, Equatorial Guinea, Gabon, Tanzania, Zambia, Zaïre, and Zimbabwe and Guyana shields (Ashton, Givnish, and Appanah 1988; Appanah 1993; Ghazoul 2016). According to Ashton (1982), Borneo has the greatest diversity of Dipterocarpaceae. Owing to economic growth, these dipterocarp forests have been heavily degraded by logging and shifting cultivation, plus converted into other land uses such as rubber and oil palm plantations (Warren‐Thomas, Dolman, and Edwards 2015; Wilcove et al. 2013).

Approximately 695 species and 16 genera have been described that belong to the Dipterocarpaceae (Christenhusz and Byng 2016). The family has a pantropical distribution and is divided into three subfamilies: Dipterocarpoideae (mainly South‐East Asia), Monotoideae (Africa and Madagascar), and Pakaraimoideae (single species *Pakaraimea dipterocarsaceae*, endemic to the Guyana Shields) (Ashton 1982, 1988; Ghazoul 2016). The source of nomenclature for the dipterocarp species used in this study was according to Symington (1974), Maguire et al. (1977), Ashton (1977, 1982, and 1988), Kostermans (1978, 1981, 1982, 1983, and 1992), and Londono et al. (1995).

Characteristically, the Dipterocarpaceae are involved in mast‐fruiting events, with synchronous intermittent (often > 7 years) production of large seed crops (Janzen 1974; Appanah 1993; Kelly and Sork 2002). Dipterocarps are pollinated by various insects during general flowering, with most dipterocarps in lowland forests being pollinated by bees, with beetles also playing a role (Momose et al. 1998), and occasionally birds (Momose et al. 1998; Sakai 2002).

### 2.2 Data Collection

We compiled plant traits data for 544 dipterocarp species from a range of resources (Table S1). These included (1) a literature search in Google Scholar with search term ‘Dipterocarpaceae’ yielding 13,400 results; (2) key monographs by Symington (1974) and Ghazoul (2016); and (3) internet plant databases (IUCN Red List), Forest Research Institute Malaysia (FRIM) website, PlantUse.net, PROSEA—Plant Resources of SouthEast Asia (https://prosea.prota4u.org/). Data that we collected for each species are (see Table S1 for details):

1. Taxonomy (sub‐family, tribe, genus, section, and sub‐section).
2. Habitat—forest habitat inhabited by dipterocarp plants (i.e., lowland forest, upper hill dipterocarp forest, and montane forest)
3. Geographic distribution: altitudinal data, estimated Extent of Occurrence, and Area of Occupancy.
4. Habitat Soil Type: We recorded the soil type inhabited by the plants.
5. Quantitative plant traits: We recorded plant height, diameter at breast height (DBH), growth rate, leaf length, mean seed weight per kilo, fruit length, fruit width, wing length, dispersal, survival, and wood density.
6. Qualitative plant traits: We recorded shade tolerance, chromosome number, flowering frequency, anthesis time, flower size, flower reward, flower color, pollinator agents, number of wings, seed dispersal agent, and wood type.
7. Threat and imperilment: We recorded conservation status (using IUCN red list status), population trend (IUCN 2018), habitat destruction, and percentage of habitat declined [Forest Research Institute Malaysia (FRIM) database; Maycock et al. 2012].

### 2.3 Phylogenetic Tree

Bansal et al. (2022) employed phylogenetic methods to trace the evolutionary history of dipterocarps. They used genetic data (fossil pollen and molecular data), and, comparing it with fossil pollen records, they aimed to reconstruct the lineage and diversification patterns of these trees. For our study, using the R package “S. PhyloMaker”, we constructed a phylogenetic tree by grafting dipterocarp genera and species included in this study onto a backbone phylogenetic hypothesis (Qian and Jin 2016). We used the PhytoPhylo mega‐phylogeny as the backbone (Qian and Jin 2016) and an updated and expanded version of Zanne et al.'s species‐level phylogeny (Zanne et al. 2014). Zanne et al.'s phylogeny comprises about 30,771 seed plants and was time‐calibrated for all branches using seven gene regions available in GenBank as well as fossil data. Moreover, PhytoPhylo includes all families of extant seed plants (Qian and Zhang 2014) with 5 times more genera and over 55 times more species than the newest angiosperm supertrees (i.e., R20120829) (Qian and Jin 2016).

For genera and species that were not found or were missing from the PhytoPhylo mega‐phylogeny, we took three approaches: (1) adding genera or species as polytomies within their families; (2) randomly adding genera or species within their families or genera; and (3) adding genera or species to their families or genera with the same approach used in the online software Phylomatic and BLADJ (Branch Length Adjuster). Using these three approaches, three phylogenies were generated at each level of resolution such as family, genus, and species (see Appendix C2).

### 2.4 Phylogenetic Niche Conservatism

As noted above, following Cooper, Freckleton, and Jetz (2011), it is important to define PNC because multiple definitions are possible. Here, we consider that PNC occurs when closely related species are similar through having inherited their niches from ancestors; conversely, PNC is absent when species traits are evolutionarily labile and there is no relationship between traits and phylogeny.

To achieve Objective 1, we calculated a phylogenetic signal for environmental factors and each plant trait in our study to measure the degree of phylogenetic conservatism across traits. We used Pagel's *λ* to identify phylogenetic dependence based on prediction of the Brownian model of trait evolution (Pagel 1999; Freckleton, Harvey, and Pagel 2002). This parameter varies between 0 and 1: *λ* = 0 suggests no phylogenetic signal, and *λ* = 1 suggests perfect phylogenetic dependence under the Brownian motion model. We estimated *λ* values for each trait by using the pgls function from R package caper (Orme 2013).

For Objective 2, we assessed the drivers of geographic distribution of dipterocarp species by using elevational gradient data and soil type as predictor variables and geographic extent and extent of occurrence as response variables in the linear model. The *λ* statistic was also used to control for phylogenetic signals in the linear models (Freckleton, Harvey, and Pagel 2002).

For Objective 3, we determined the relationship between morphological traits (e.g., height and DBH) and species performance (i.e., growth and survival) as response variables with habitat soil type and shade tolerance traits as predictor variables.

Finally, for Objective 4, we used conservation status as the response variable and population trends and habitat destruction as predictor variables in the linear model to assess correlation between conservation status and phylogeny in the Dipterocarpaceae family, as well as to explore whether PNC contributes to extinction threats. These linear models were fitted by using the pgls function in caper package in R software.

## 3 Results

### 3.1 Phylogenetic Signal in Individual Traits

Lower and upper elevation limit showed phylogenetic dependence, with values of *λ* 0.675 and 0.468, respectively (*p* < 0.001 for tests of *λ* = 0, Table 1), indicating some degree of phylogenetic conservatism in altitudinal preferences. The geographic range of distribution in dipterocarp species showed weak phylogenetic dependence with the *λ* value of 0.048 (*p* < 0.05 for *λ* = 1; Table 1). However, no phylogenetic signal was observed in the estimated area of occupancy (*p* ns for *λ* = 0; ns for *λ* = 1).

**TABLE 1:** Pagel's lambda value based on the dipterocarp phylogenetic tree in a model for single trait only.

Trait (*y*)	*n*	*y* ~ 1		
		*λ*	*P*(*λ* = 0)	*P*(*λ* = 1)
*Elevation*				
Lower elevation limit (m)	523	0.675	***	***
Upper elevation limit (m)	523	0.468	***	***
*Geographic distribution*				
Widespread/endemic	541	0.216	***	***
Estimated extent of occurrence	172	0.048	*	***
Estimated area of occupancy	11	0.000	ns	ns
*Habitat Soil type*				
Soil type (Clay)	310	0.196	*	***
Soil type (Sandy)	310	0.263	**	***
Soil type (Loam)	310	0.456	***	***
Soil type (Limestone)	310	0.000	ns	***
*Morphological traits*				
Height	387	0.547	***	***
Diameter at breast height	353	0.410	***	***
Growth rate	29	0.758	ns	ns
Shade tolerance	241	0.732	***	***
Leaf length (cm)	381	0.216	*	***
Flower size (mm)	392	0.831	***	***
Flower reward (Nectar)	323	0.221	*	***
Flower reward (Pollen)	323	0.122	***	***
Flower reward (Corolla)	323	0.047	**	***
Survival (%)	51	0.000	ns	***
*Flowering event*				
Flowering frequency	543	0.687	***	***
Anthesis (Day)	142	0	ns	***
Anthesis (Night)	142	1	***	ns
*Genetic traits*				
Chromosome no. (*x* = 7)	544	1	***	ns
Chromosome no.(*x* = 10)	544	1	***	ns
Chromosome no.(*x* = 11)	544	1	***	ns
Polyploidy	544	1	***	ns
Outcrossing rate	19	0	ns	***
*Seed traits*				
Fruit length (mm)	282	0.500	***	***
Fruit width (mm)	239	0.383	**	***
Seed weight (seed per kilo)	65	0.996	***	ns
Wingless seed	543	0.505	***	***
Functional wing = 2	543	1	***	ns
Functional wing = 3	543	1	***	ns
Functional wing = 5	543	1	***	ns
Functional wing length	111	0.167	**	***
Wing loading	25	0.000	ns	*
*Timber type and density*				
Wood type	484	0.841	***	***
Wood densities	238	0.442	***	***

Abbreviation: ns, not significant.

*p < 0.05; **p < 0.01; ***p < 0.001.

Of the morphological traits, plant height, diameter at breast height (DBH), flower size, flower reward, and shade tolerance showed phylogenetic dependence with *λ* values ranging from 0.41 to 0.831 (all *p* < 0.001 for tests of *λ* = 0; Table 1). Leaf length and flower reward nectar traits were significantly conserved in dipterocarp species with *λ* values of 0.216 and 0.221, respectively (*p* < 0.05 in *λ* = 0; Table 1). Survival showed phylogenetic independence (*p* ns for *λ* = 0; < 0.001 for *λ* = 1). Flowering frequency in dipterocarp species showed phylogenetic dependence with *λ* of 0.687 (*p* < 0.001 in *λ* = 0; Table 1). For all genetic traits, there was phylogenetic dependence in dipterocarp species with all *λ* value of 1 except for the outcrossing rate (*p* < 0.001 in *λ* = 0; Table 1).

In terms of seed traits, both fruit length and wingless seed exhibited phylogenetic dependence with *λ* values of 0.500 and 0.505, respectively (*p* < 0.001 in *λ* = 0; Table 1). Seed weight showed a phylogenetic signal with a high *λ* value of 0.996 (*p* < 0.001 in *λ* = 0; Table 1). Functional wing length and fruit width had *λ* of 0.167 and 0.383 (*p*, 0.05 for *λ* = 0). In addition, timber type and wood densities showed phylogenetic dependence with *λ* values of 0.841 and 0.442, respectively (all *p* < 0.001 in *λ* = 0; Table 1).

### 3.2 Drivers of Geographic Distribution

**FIGURE 1:**
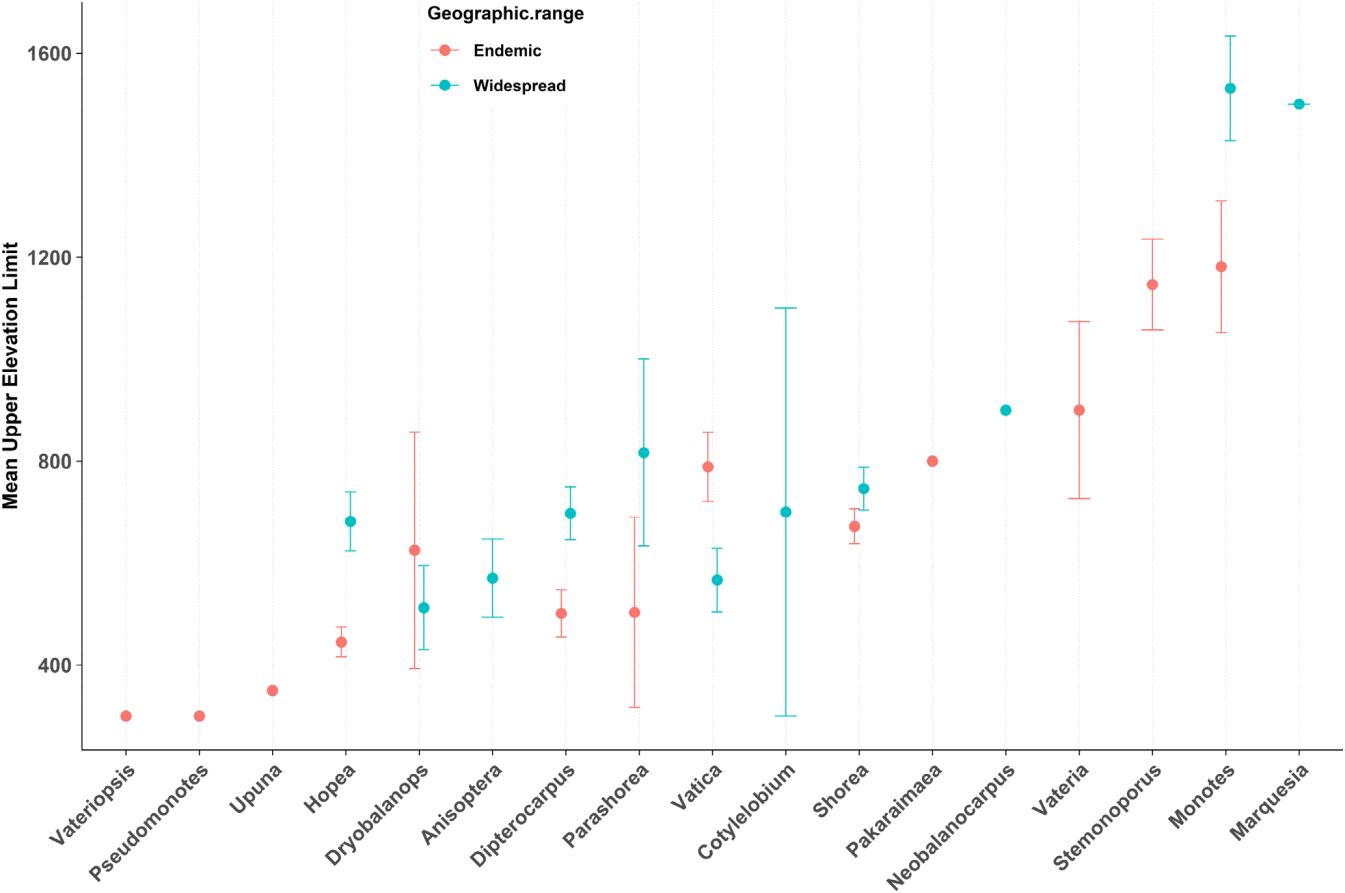
Relationship of the upper elevation limit of each dipterocarp genera between the geographic range. Total species—544, number of species in each genera; vateriopsis—1; Pseudomonotes—1; Upuna—1; Hopea—113; Dryobalanops—8; Anisoptera—10; Dipterocarpus—67; Parashorea—14; Vatica—75; Cotylelobium—5; Shorea—192; Pakaraimaea—1; Neobalanocarpus—1; Vateria—3; Stemonoporus—26; Monotes—23; Marquesia—3.

The upper elevation limit showed significant association with species distribution with weak phylogenetic dependence (*λ* = 0.227, *p* < 0.001 in *λ* = 0; Table 2; Figure 1). In all models for distribution versus elevational gradient, there were weak phylogenetic signals with *λ* values ranging from 0.202 to 0.227 (*p* < 0.001 for both *λ* = 0 and *λ* = 1; Table 2). The upper elevation limit exhibited a significant relationship with the estimated extent of species occurrence but showed no phylogenetic dependence (*p* ns for *λ* = 0; Table 2).

**TABLE 2:** *F* and *λ* values for phylogenetic linear models testing the relationships between (a) species distribution and elevational gradient; (b) extent of occurrence and elevational gradient.

	*n*	Elevational gradient (*F* ^*P*^)		*λ*	*P*(*λ* = 0)	*P*(*λ* = 1)
		Lower limit (lwr)	Upper limit (upr)			
*(a) Geographic extent*						
Distribution~ lwr	519	0.073^ns^		0.207	***	***
Distribution~ upr	519		6.423*	0.227	***	***
Distribution ~ lwr + upr	518	0.080^ns^	10.268**	0.202	***	***
*(b) Extent of occurrence (EOO)*						
EOO ~ lwr	171	0.001^ns^		0.048	*	***
EOO ~ upr	171		9.516**	0.048	ns	***
EOO ~ lwr + upr	170	0.001^ns^	11.529***	0.044	ns	***

Abbreviation: ns, not significant.

*p < 0.05; **p < 0.01; ***p < 0.001.

Of the variables measuring soil types, only the presence of limestone showed a significant effect on species distribution (Table 3), with a weak phylogenetic signal (*λ* = 0.275, *p* < 0.001 in *λ* = 0; Table 3). There was a significant relationship between limestone soil type and estimated extent of occurrence (Table 3), but none of all these soil types showed phylogenetic dependence (all *p* ns for *λ* = 0; Table 3).

**TABLE 3:** *F* and *λ* values for phylogenetic linear models testing the relationships between (a) species distribution and habitat soil types; (b) extent of occurrence and habitat soil types.

	*n*	Soil type (*F* ^*P*^)				*λ*	*P*(*λ* = 0)	*P*(*λ* = 1)
		Clay (c)	Sandy (s)	Loam (l)	Limestone (ls)			
*(a) Geographic extent*								
Distribution ~ Clay	307	3.130^ns^				0.300	***	***
Distribution ~ Sandy	307		1.005^ns^			0.278	***	***
Distribution ~ Loam	307			1.220^ns^		0.282	***	***
Distribution ~ Limestone	307				5.104*	0.275	***	***
Distribution ~ c + s + l + ls	304	3.112^ns^	0.059^ns^	0.883^ns^	4.375*	0.288	***	***
*(b) Extent of occurrence (EOO)*								
EOO ~ Clay	110	0.195^ns^				0.000	ns	***
EOO ~ Sandy	110		0.016^ns^			0.000	ns	***
EOO ~ Loam	110			0.403^ns^		0.000	ns	***
EOO ~ Limestone	110				20.221***	0.052	ns	***
EOO ~ c + s + l + ls	107	0.304^ns^	0.006^ns^	0.591^ns^	21.003***	0.066	ns	***

Abbreviation: ns, not significant.

*p < 0.05; **p < 0.01; ***p < 0.001.

### 3.3 Morphological Traits and Habitat Factors

Based on our results, clay soil type showed a significant relationship with tree height, although there was no association of height with other soil types or shade tolerance (Table 4a), with significant phylogenetic dependence (*λ* = 0.533, *p* < 0.001; Table 4a). Meanwhile, habitat soil type such as clay and sandy exhibited significant association with tree diameter (Table 4b) and mild phylogenetic dependence with *λ* values of 0.450 and 0.427, respectively (*p* < 0.001 in *λ* = 0; Table 4).

**TABLE 4:** *F* and *λ* values for a phylogenetic linear model testing the relationship between morphological traits on soil types and shade tolerance.

	*n*	*F* ^*P*^	*λ*	*P*(*λ* = 0)	*P*(*λ* = 1)
*(a) Height*					
Clay	289	6.697**	0.533	***	***
Sandy	289	2.938^ns^	0.516	***	***
Loam	289	0.120^ns^	0.543	***	***
Limestone	289	0.024^ns^	0.542	***	***
Shade tolerance	171	0.416^ns^	0.582	***	***
*(b) Diameter at breast height (DBH)*					
Clay	273	5.072*	0.450	***	***
Sandy	273	4.566*	0.427	***	***
Loam	273	0.833^ns^	0.473	***	***
Limestone	273	0.627^ns^	0.470	***	***
Shade tolerance	162	0.136^ns^	0.504	***	***
*(c) Growth*					
Clay	20	0.146^ns^	0.392	ns	ns
Sandy	20	0.715^ns^	0.264	ns	ns
Loam	20	0.001^ns^	0.433	ns	ns
Limestone	20	3.046^ns^	0.858	ns	ns
Shade tolerance	21	0.108^ns^	0.490	ns	ns
*(d) Survival*					
Clay	37	1.855^ns^	0.000	ns	***
Sandy	37	0.727^ns^	0.000	ns	***
Loam	37	1.018^ns^	0.000	ns	***
Limestone	37	0.028^ns^	0.000	ns	***
Shade tolerance	31	5.314*	0.000	ns	**

Abbreviation: ns, not significant.

*p < 0.05; **p < 0.01; ***p < 0.001.

**FIGURE 2:**
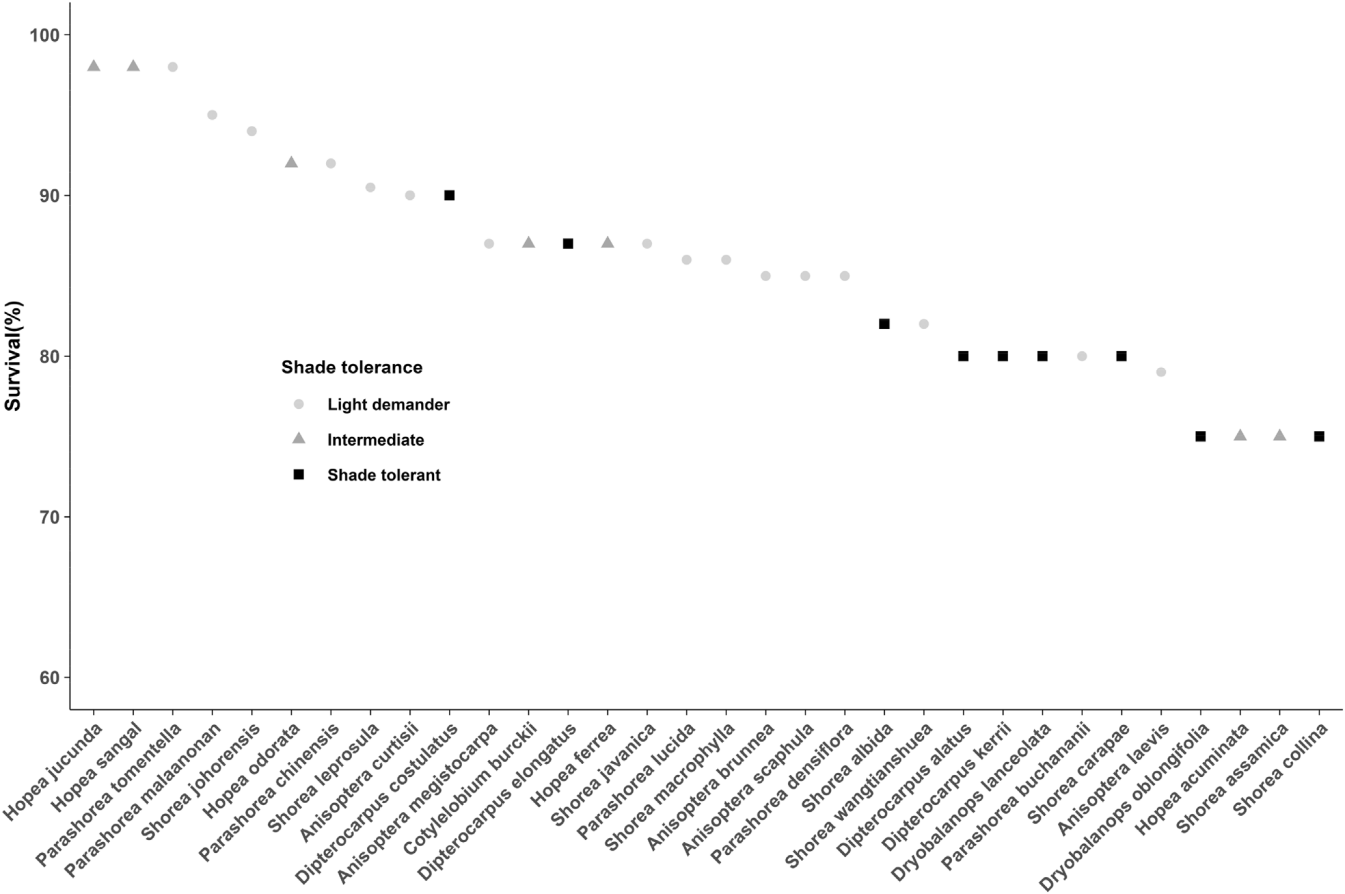
Survival rate of 32 dipterocarp species based on their shade tolerance trait.

In all the models of growth versus soil types and shade tolerance, there were no significant relationships (Table 4c). Furthermore, no phylogenetic signals were observed in all growth versus soil types and shade tolerance models, with *λ* values not distinguishable from either 0 or 1. When survival was modeled against soil types and shade tolerance, only shade tolerance traits showed a statistically significant association with survival (Table 4d; Figure 2). However, none of the models showed phylogenetic dependence (all *p* ns for *λ* = 0; Table 4d).

### 3.4 Conservation Status and Phylogeny

**FIGURE 3:**
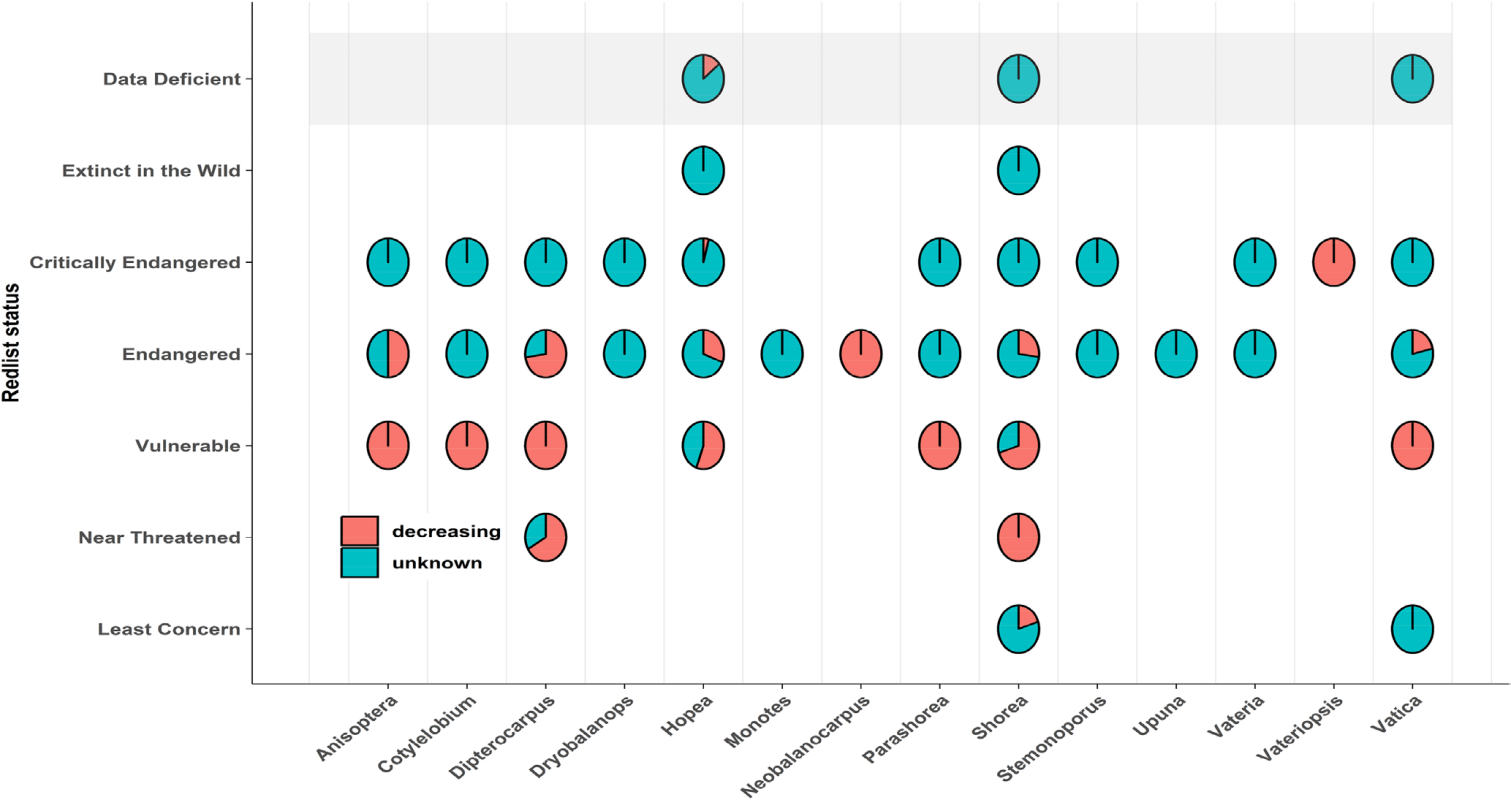
Population trends of dipterocarp genera based on the IUCN Red List status.

**FIGURE 4:**
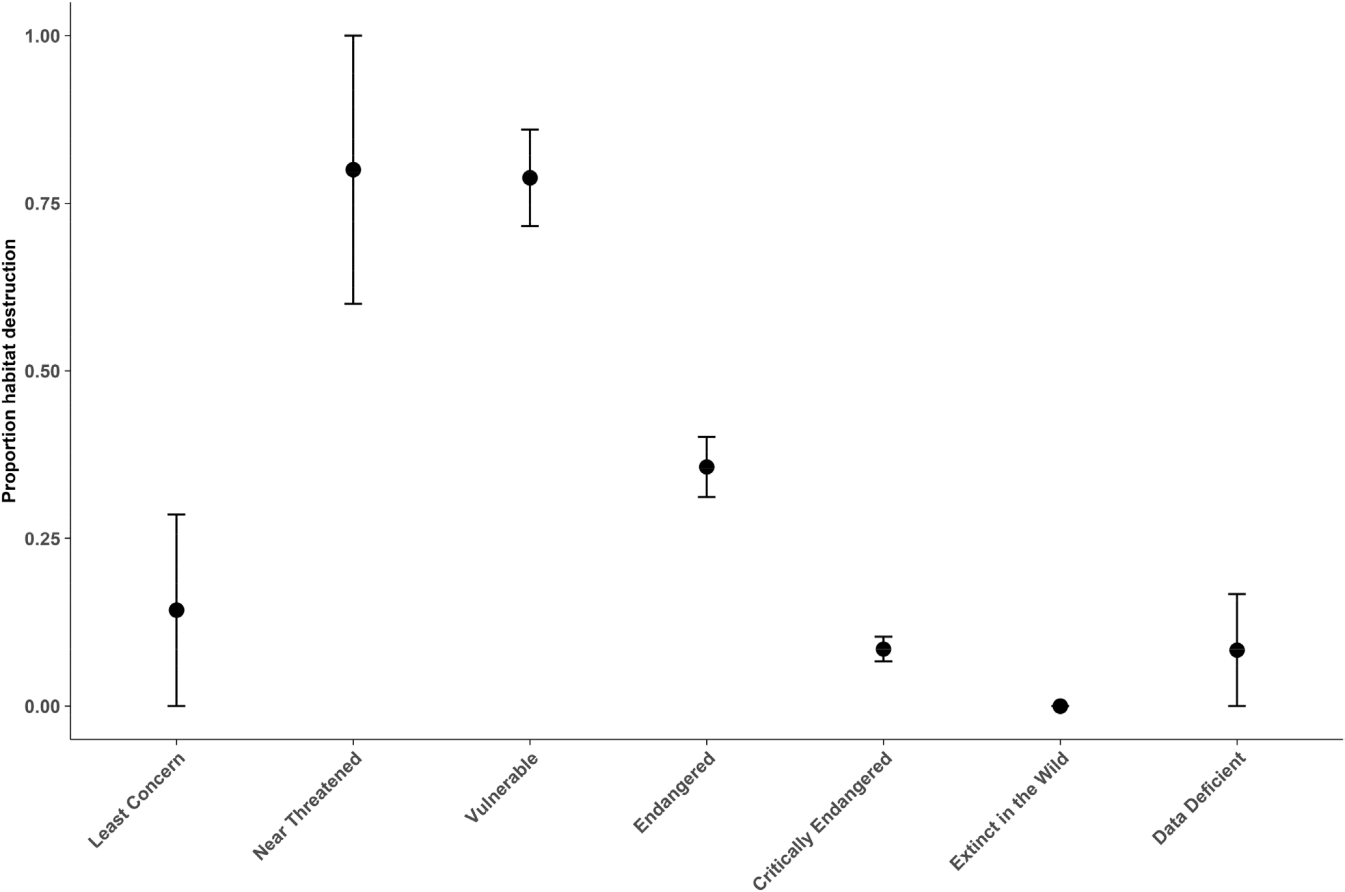
Relationship between habitat destruction and IUCN Red List status.

**FIGURE 5:**
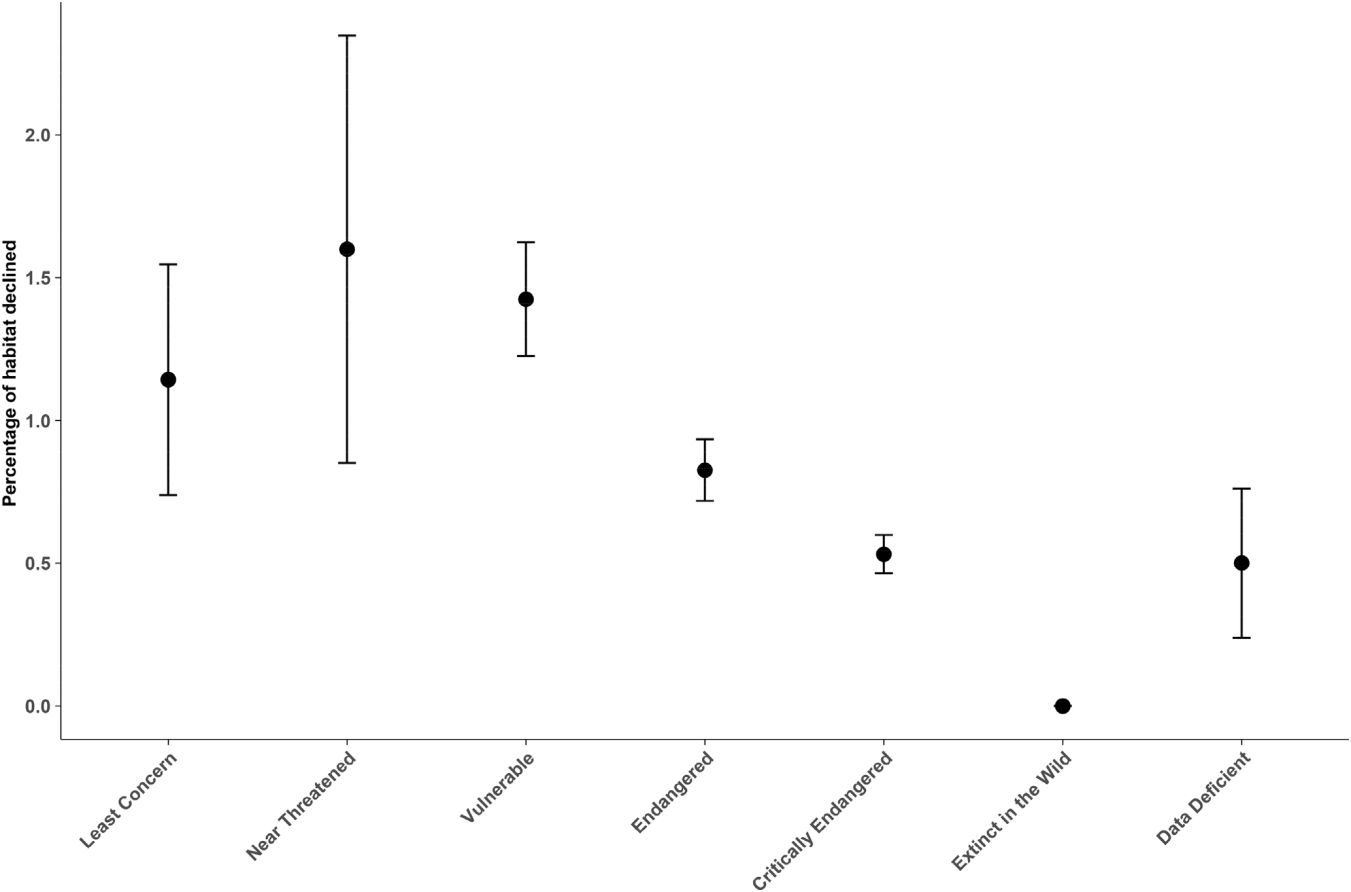
Relationship of percentage of habitat declined with IUCN Red List status. Unknown percentage = 0, Less than 30% = 1, Less than 50% = 2, Less than 80% = 3, More than 80% = 4).

The species population trend exhibited a significant relationship with conservation status (Table 5; Figure 3), with strong evidence for phylogenetic dependence (*λ* = 0.536, *p* < 0.001 in *λ* = 0; Table 5; Figure 3). Furthermore, habitat destruction and percentage of habitat decline also showed significant association with conservation status but was not related to phylogeny (both *p* ns for *λ* = 0; Table 5; Figures 4 and 5).

**TABLE 5:** *F* and *λ* values for phylogenetic linear models testing the relationship between conservation status on population trend and habitat destruction.

	*n*	*F* ^*P*^	*λ*	*P*(*λ* = 0)	*P*(*λ* = 1)
*Red list status*					
Population trend	397	75.287***	0.536	***	***
Habitat destruction	397	34.812***	0.401	ns	***
Percentage of habitat decline	397	8.984**	0.000	ns	***

Abbreviation: ns, not significant.

*p < 0.05; **p < 0.01; ***p < 0.001.

## 4 Discussion

### 4.1 PNC in Dipterocarp Species Traits

Our results revealed that there were significant phylogenetic signals in most of the plants traits we measured. This phylogenetic signal is consistent with PNC in these traits, defined as a tendency of closely related species to exhibit similar trait values. PNC in Dipterocarpaceae might be associated with a suite of different evolutionary processes with implications for our understanding of biogeography and the future impacts of climate change (Wiens and Graham 2005).

The phylogenetic associations of dipterocarps have been difficult to uncover. Reasons include hybridization between species and interspecific heterogeneity in molecular and morphological traits (Ashton 1988; Dayanandan et al. 1999; Gamage et al. 2006). In the *Shorea* genus, for example, interspecific hybridization is known in aseasonal rainforest in Malaysia and Indo‐Burma (Ashton 1982; Ishiyama et al. 2008; Kamiya et al. 2011). The evolutionary history of this group is complex, but the existence of a phylogenetic signal in a suite of traits indicates that despite this complexity, some conservatism in traits exists. The obvious question, therefore, is whether this impacts distributions or population status in terms of conservation or population trends.

### 4.2 Environmental Adaptations and Species Distribution

We found evidence that the upper elevation limits of dipterocarp species are related to distribution (widespread species have wider limits). Many dipterocarp species are restricted to lowland forest, and species richness gradually decreases from above ~400 m above sea level. For instance, dipterocarp species in Sumatra, such as *Shorea pauciflora, Shorea macroptera, Shorea singkawang*, and *Anisoptera megistocarpa*, are strictly limited to elevations up to 200 m (Ghazoul 2016). To date, there is little information on how elevational gradients have influenced plant endemism (Kessler 2002). Our results showed that upper elevations limit species' extent of occurrence, but this does not relate to phylogeny. This suggests that limits on extent of occurrence affect all species and do not affect different clades differentially.

Our analyses demonstrated that the soil types inhabited by species are related to phylogeny, suggesting that dipterocarp clades have undergone evolutionary adaptation to edaphic types. This is supported by Ashton and Ashton (2014), who found that the occurrence of dipterocarps in Borneo is related to particular soil types. Tropical forest soils are heterogenous, and the most common soil types in tropical forests are ultisols and oxisols, which are highly weathered clay soils with low nutrient content and pH value (Shamshuddin and Fauziah, 2010; Ghazoul 2016). Only limestone soil type showed a significant relationship with distribution and species extent of occurrence. This result may be due to lack of soil type information in our data, although limestone soils (alfisols) are rich in nutrients but are less common (Potts et al. 2002; Ghazoul 2016). Several studies stated that the role of symbiont mycorrhiza on the limestone area could affect species distribution due to different assemblage of ‎ectomycorrhiza may be required on alkaline limestone soils as opposed to other more acidic soil types (Johnson, Liu, and Burslem 2023; Rineau and Garbaye 2009).

### 4.3 Morphological Traits, Habitats, and Life History Strategies

Our results suggest that habitat soil types showed significant relationships with tree height and diameter, particularly in clay and sandy soil types. Soil plays a significant role in shaping dipterocarp forest communities and plant growth (Paoli, Curran, and Zak 2006). Many tropical species especially dipterocarp grow in highly weathered clay soils, which are acidic and have low nutrient content (Chazdon 2003; Palmiotto et al. 2004; Paoli, Curran, and Zak 2006; Peay et al. 2010). However, the presence of humus content and ectomycorrhizal fungi association in dipterocarp species contributed to tree height and diameter growth by enhancing and retaining the nutrient uptake (Brearley, Press, and Scholes 2003; Ducousso et al. 2004; Baillie et al. 2006; Paoli, Curran, and Zak 2006; Tedersoo et al. 2007).

Our data showed that survival was associated with shade tolerance traits, but there was no imprint of phylogeny. Shade‐tolerant species show high survival, potentially for many years. For instance, a long‐term study by Delissio et al. (2002) found that between 38% and 61% of seedlings of four shade‐tolerant dipterocarps (*Cotylebium melanoxylon, Dipterocarpus globosus, Dryobalanops beccarii, and Shorea beccariana*) survived over a decade in the understory. However, shade‐tolerant dipterocarp species unable to capitalize available light in canopy gap as light‐demanding and intermediate species. Shade‐tolerant species like most dipterocarp species thrive in low‐light environments, using adaptations that allow them to efficiently photosynthesize and grow slowly, while light‐demanding species require full sunlight to flourish, exhibiting traits that maximize growth and photosynthesis in bright conditions (Barker, Press, and Brown 1997; Widiyatno et al. 2020). These differences highlight their distinct strategies for survival and competition in various ecosystems. Our results emphasized that the plant life‐history strategies traits influence the species' survival.

### 4.4 Conservation Status of Dipterocarp

Timber exploitation of dipterocarps, which have high commercial value, is a major driver of their decline in tropical forests. Furthermore, some dipterocarps like *Dipterocarpus lamellatus* and *Shorea blumutensis* occur in small population sizes and limited geographic distributions, suggesting that they could be heavily affected by exploitation and habitat loss and thus threatened by extinction (Yeong, Reynolds and Hill, 2016). Our findings revealed that the population trend influenced conservation status in dipterocarp species. This pattern was related to phylogeny, with the results showing a moderate phylogenetic signal. This is supported by various studies that found extinction risk is correlated with phylogeny (Purvis et al. 2005; Sjöström and Gross 2006; Willis et al. 2008; Fritz and Purvis 2010).

Understanding evolutionary history in deciding conservation priorities could maximize conservation of biodiversity (Faith 1992; Faith, Reid, and Hunter 2004; Redding and Mooers 2006). In our analysis, habitat destruction (i.e., logging, urbanization, and agricultural plantation) and loss of extensive habitat are major factors affecting the conservation status of dipterocarps. Due to their high value in the global timber trade and rapid deforestation for oil palm and rubber, dipterocarp timbers face threats from habitat destruction and timber exploitation. For example, in Sabah, the projected percentage of habitat loss was 21% for *Shorea micans* and 99.5% for *Dipterocarpus lamellatus*, suggesting that 32 of the 33 dipterocarp species studied would have been classified as “Threatened” under IUCN Red List criteria (Maycock et al. 2012). Our findings highlighted the conservation priorities of dipterocarp in the future for sustainable forest.

Dipterocarps, primarily native to Southeast Asia, are also found in tropical regions of India and Africa, where they play an important ecological and economic role. In India, species like *Shorea robusta* are prominent in the Western Ghats and northeastern forests, contributing to timber production, resin harvesting, and biodiversity (Nandy, Ghosh, and Singh 2021; Sahoo et al. 2024). These trees are vital for maintaining tropical forest structure, stabilizing soils, and supporting wildlife. However, over‐exploitation of timber, logging, and habitat loss due to deforestation pose significant threats to their survival in the region. In Africa, while true Dipterocarps are less common, genus like Marquesia and Monotes play a similar ecological role in West and Central African rainforests, providing timber and resin for local use, but are also at risk due to deforestation (Meerts, Rougelot, and Sosef 2017).

Despite their different geographical distributions, dipterocarps in both regions are essential for forest ecosystems, contributing to nutrient cycling and forest canopy structure. In India, the economic value of dipterocarps is more significant due to the scale of timber industries and resin collection, while in Africa, these species are less commercially exploited but still vital for local communities (Ghazoul 2016). Conservation concerns in both regions arise from habitat destruction, illegal logging, and agricultural expansion, which threaten these trees. Efforts to protect dipterocarp forests are crucial to maintaining the biodiversity and ecological services they provide.

## 5 Conclusion

We found substantial evidence of phylogenetic conservatism of plant traits in dipterocarp species, with a moderate phylogenetic signal in our results. Our findings showed that elevational gradients are involved in shaping dipterocarp species distribution across the range of the group. Morphological traits such as height and diameter show phylogenetically dependent relationship with the habitat soil types. This study highlighted the significance of plant traits analysis and revealed the association between plant traits and environmental factors pan‐tropically. However, taxonomy of dipterocarps remains challenging at some levels. One limitation in this study is that there was no complete phylogeny for all 544 dipterocarp species. Notwithstanding that, phylogenetic analysis seems to be a powerful tool to highlight conservation priorities in dipterocarp biodiversity, since phylogenies provide an additional measure of biodiversity that complements species richness. Considering evolutionary distinctiveness should play a role in prioritizing species for conservation.

## Author Contributions

**Nazrin Malik:** conceptualization (equal), data curation (equal), formal analysis (equal), investigation (lead), methodology (equal), project administration (equal), validation (equal), visualization (lead), writing – original draft (lead). **David Edwards:** conceptualization (equal), project administration (equal), supervision (supporting), validation (equal), visualization (equal), writing – review and editing (equal). **Robert P. Freckleton:** conceptualization (equal), data curation (equal), formal analysis (equal), methodology (equal), project administration (equal), supervision (lead), validation (equal), visualization (equal), writing – review and editing (equal).

## Conflicts of Interest

The authors declare no conflicts of interest.

## Supporting information


Appendix S1.

Data S1.

Data S2.

Table S1.

## Data Availability

The data that support the findings of this study has been provided in the Supporting Information.

## References

[ece370784-bib-0001] Ackerly, D. D. 2004. “Adaptation, Niche Conservatism, and Convergence: Comparative Studies of Leaf Evolution in the California Chaparral.” American Naturalist 163, no. 5: 654–671. 10.1086/383062 15122485

[ece370784-bib-0002] Appanah, S. 1993. “Mass Flowering of Dipterocarp Forests in the Aseasonal Tropics.” Journal of Biosciences 18, no. 4: 457–474.

[ece370784-bib-0003] Appanah, S. , and J. M. Turnbull . 1998. A Review of Dipterocarps: Taxonomy, Ecology and Silviculture. Bogor, Indonesia: Center for International Forestry Research.

[ece370784-bib-1009] Ashton, P. S. 1977. “Dipterocarpaceae.” In Revised Handbook to the Flora of Ceylon, edited by B. A. Abeywickrama , vol. 1, 166–196. New Delhi: Amerind Publ. Co.

[ece370784-bib-0004] Ashton, P. 1982. “Dipterocarpaceae.” https://www.cabdirect.org/cabdirect/abstract/19820681886.

[ece370784-bib-0005] Ashton, P. S. 1988. “Dipterocarp Biology as a Window to the Understanding of Tropical Forest Structure.” Annual Review of Ecology and Systematics 19: 347–370.

[ece370784-bib-0006] Ashton, P. S. , T. J. Givnish , and S. Appanah . 1988. “Staggered Flowering in the Dipterocarpaceae: New Insights Into Floral Induction and the Evolution of Mast Fruiting in the Aseasonal Tropics.” American Naturalist 132, no. 1: 44–66.

[ece370784-bib-1019] Ashton, P. , and M. Ashton . 2014. “Guest Editorial: Mixed Dipterocarp Forests of the Sunda Lands: What Can Be Done Now with What Is Left?” Journal of Tropical Forest Science 26, no. 2: 163–165. http://www.jstor.org/stable/23723900.

[ece370784-bib-0008] Baillie, I. C. , P. S. Ashton , S. P. Chin , et al. 2006. “Spatial Associations of Humus, Nutrients and Soils in Mixed Dipterocarp Forest at Lambir, Sarawak, Malaysian Borneo.” Journal of Tropical Ecology 22, no. 5: 543–553.

[ece370784-bib-1007] Bansal, M. , R. J. Morley , S. K. Nagaraju , et al. 2022. “Southeast Asian Dipterocarp Origin and Diversification Driven by Africa‐India Floristic Interchange.” Science 375, no. 6579: 455–460. 35084986 10.1126/science.abk2177

[ece370784-bib-0009] Barker, M. G. , M. C. Press , and N. D. Brown . 1997. “Photosynthetic Characteristics of Dipterocarp Seedlings in Three Tropical Rain Forest Light Environments: A Basis for Niche Partitioning?” Oecologia 112: 453–463. 28307621 10.1007/s004420050332

[ece370784-bib-0010] Blomberg, S. P. , T. Garland , and A. R. Ives . 2003. “Testing for Phylogenetic Signal in Comparative Data: Behavioral Traits Are More Labile.” Evolution 57, no. 4: 717–745. 12778543 10.1111/j.0014-3820.2003.tb00285.x

[ece370784-bib-0011] Blomberg, S. P. , J. G. Lefevre , J. A. Wells , and M. Waterhouse . 2012. “Independent Contrasts and PGLS Regression Estimators are Equivalent.” Systematic Biology 61, no. 3: 382–391. 22215720 10.1093/sysbio/syr118

[ece370784-bib-0013] Brearley, F. Q. , M. C. Press , and J. D. Scholes . 2003. “Nutrients Obtained From Leaf Li Tter Can Improve The Growth Of Dipterocarp Seedlings Nutrisi Yang Diperoleh Dari Serasah Daun Meningkatkan Pertumbuhan Semai Dipterocarpaceae Ringkasan Pendahuluan.” Area 160: 101–110. 10.1046/j.1469-8137.2003.00851.x 33873536

[ece370784-bib-0014] Chazdon, R. L. 2003. “Tropical Forest Recovery: Legacies of Human Impact and Natural Disturbances.” Perspectives in Plant Ecology, Evolution and Systematics 6, no. 1–2: 51–71.

[ece370784-bib-0016] Christenhusz, M. J. M. , and J. W. Byng . 2016. “The Number of Known Plants Species in the World and Its Annual Increase.” Phytotaxa 261, no. 3: 201–217.

[ece370784-bib-1004] Comita, L. S. , S. A. Queenborough , S. J. Murphy , et al. 2014. “Testing Predictions of the Janzen–Connell Hypothesis: A Meta‐Analysis of Experimental Evidence for Distance‐and Density‐Dependent Seed and Seedling Survival.” Journal of Ecology 102, no. 4: 845–856. 25253908 10.1111/1365-2745.12232 PMC4140603

[ece370784-bib-0017] Connell, J. H. 1971. “On the Role of Natural Enemies in Preventing Competitive Exclusion in Some Marine Animals and in Rain Forest Trees.” In Dynamics of populations. Proceeding of the Advanced Study Institute, edited by P. J. den Boer and G. Gradwell , 298–312. Osterbeek: Centre for Agricultural Publishing and Documentation.

[ece370784-bib-0018] Cooper, N. , R. P. Freckleton , and W. Jetz . 2011. “Phylogenetic Conservatism of Environmental Niches in Mammals.” Proceedings of the Royal Society B: Biological Sciences 278, no. 1716: 2384–2391. 10.1098/rspb.2010.2207 PMC3119006 21208967

[ece370784-bib-0019] Cooper, N. , G. H. Thomas , and R. G. FitzJohn . 2016. “Shedding Light on the ‘Dark Side’ of Phylogenetic Comparative Methods R. B. O'Hara, Ed.” Methods in Ecology and Evolution 7, no. 6: 693–699. 27499839 10.1111/2041-210X.12533 PMC4957270

[ece370784-bib-0021] Dayanandan, S. , P. S. Ashton , S. M. Williams , and R. B. Primack . 1999. “Phylogeny of the Tropical Tree Family Dipterocarpaceae Based on Nucleotide Sequences of the Chloroplast rbcL Gene.” American Journal of Botany 86, no. 8: 1182–1190. 10449398

[ece370784-bib-1023] Delissio, L. J. , R. B. Primack , P. Hall , and H. S. Lee . 2002. “A Decade of Canopy‐Tree Seedling Survival and Growth in Two Bornean Rain Forests: Persistence and Recovery from Suppression.” Journal of Tropical Ecology 18, no. 5: 645–658.

[ece370784-bib-0024] Ducousso, M. , G. Béna , C. Bourgeois , et al. 2004. “The Last Common Ancestor of Sarcolaenaceae and Asian Dipterocarp Trees Was Ectomycorrhizal Before the India‐Madagascar Separation, About 88 Million Years Ago.” Molecular Ecology 13, no. 1: 231–236. 14653803 10.1046/j.1365-294x.2003.02032.x

[ece370784-bib-0025] Faith, D. P. 1992. “Conservation Evaluation and Phylogentic Diversity.” Biological Conservation 61: 1–10.

[ece370784-bib-0026] Faith, D. P. , C. A. M. Reid , and J. Hunter . 2004. “Integrating Phylogenetic Diversity, Complementarity, and Endemism for Conservation Assessment.” Conservation Biology 18, no. 1: 255–261.

[ece370784-bib-0028] Freckleton, R. P. 2009. “The Seven Deadly Sins of Comparative Analysis.” Journal of Evolutionary Biology 22, no. 7: 1367–1375. 19508410 10.1111/j.1420-9101.2009.01757.x

[ece370784-bib-0029] Freckleton, R. P. , P. H. Harvey , and M. Pagel . 2002. “Phylogenetic Analysis and Comparative Data: A Test and Review of Evidence.” American Naturalist 160, no. 6: 712–726. 10.1086/343873 18707460

[ece370784-bib-0030] Fritz, S. A. , and A. Purvis . 2010. “Selectivity in Mammalian Extinction Risk and Threat Types: A New Measure of Phylogenetic Signal Strength in Binary Traits.” Conservation Biology 24, no. 4: 1042–1051. 20184650 10.1111/j.1523-1739.2010.01455.x

[ece370784-bib-0031] Gamage, D. T. , M. P. Silva , N. Inomata , T. Yamazaki , and A. E. Szmidt . 2006. “Comprehensive Molecular Phylogeny of the Sub‐Family Dipterocarpoideae (Dipterocarpaceae) Based on Chloroplast DNA Sequences.” Genes & Genetic Systems 81, no. 1: 1–12. 16607036 10.1266/ggs.81.1

[ece370784-bib-0032] Ghazoul, J. 2016. “Dipterocarp Biology, Ecology, and Conservation.” https://books.google.co.uk/books?hl=en&lr=&id=NX4eDQAAQBAJ&oi=fnd&pg=PP1&dq=jaboury+ghazoul+dipterocarp++biology+&ots=lVqwvuFHTR&sig=ZdtUNA9reG6NtkvVRkQVVi5Per0.

[ece370784-bib-0034] Harvey, P. H. , and M. D. Pagel . 1991. “The Comparative Method in Evolutionary Biology.” Trends in Ecology & Evolution 239, no. 3: 239.

[ece370784-bib-0036] Hubbell, S. P. 2001. The Unified Neutral Theory of Biodiversity and Biogeography. United Kingdom: Princeton University Press.

[ece370784-bib-0037] Ishiyama, H. , N. Inomata , T. Yamazaki , N. A. A. Shukor , and A. E. Szmidt . 2008. “Demographic History and Interspecific Hybridization of Four Shorea Species (Dipterocarpaceae) From Peninsular Malaysia Inferred From Nucleotide Polymorphism in Nuclear Gene Regions.” Canadian Journal of Forest Research 38, no. 5: 996–1007.

[ece370784-bib-1016] IUCN . 2018. The IUCN Red List of Threatened Species. Version 2018‐2. https://www.iucnredlist.org.

[ece370784-bib-0039] Janzen, D. 1974. “Tropical Blackwater Rivers, Animals, and Mast Fruiting by the Dipterocarpaceae.” Biotropica 6, no. 2: 69–103.

[ece370784-bib-0040] Janzen, D. H. 1970. “Herbivores and the Number of Tree Species in Tropical Forests.” American Naturalist 104, no. 940: 501–528.

[ece370784-bib-0041] Johnson, D. , X. Liu , and D. F. Burslem . 2023. “Symbiotic Control of Canopy Dominance in Subtropical and Tropical Forests.” Trends in Plant Science 28, no. 9: 995–1003. 37087357 10.1016/j.tplants.2023.03.027

[ece370784-bib-0044] Kamiya, K. , Y. Y. Gan , S. K. Y. Lum , M. S. Khoo , S. C. Chua , and N. N. H. Faizu . 2011. “Morphological and Molecular Evidence of Natural Hybridization in Shorea (Dipterocarpaceae).” Tree Genetics & Genomes 7, no. 2: 297–306.

[ece370784-bib-0045] Katabuchi, M. , H. Kurokawa , S. J. Davies , S. Tan , and T. Nakashizuka . 2012. “Soil Resource Availability Shapes Community Trait Structure in a Species‐Rich Dipterocarp Forest.” Journal of Ecology 100, no. 3: 643–651.

[ece370784-bib-0046] Kelly, D. , and V. L. Sork . 2002. “Mast Seeding in Perennial Plants: Why, How, Where?” Annual Review of Ecology and Systematics 33, no. 1: 427–447.

[ece370784-bib-0048] Kessler, M. 2002. “The Elevational Gradient of Andean Plant Endemism: Varying Influences of Taxon‐Specific Traits and Topography at Different Taxonomic Levels.” Journal of Biogeography 29, no. 9: 1159–1165.

[ece370784-bib-1010] Kostermans, A. J. G. H. 1978. “ *Pakaraimaea dipterocarpacea* Maguire and Ashton Belongs to Tiliaceae and Not to Dipterocarpaceae.” Taxon 27: 357–359.

[ece370784-bib-1011] Kostermans, A. J. G. H. 1981. “The Ceylonese Species of Balanocarpus Bedd. (Dipterocarpaceae).” Bulletin du Muséum National d’Histoire Naturelle, Adansonia, 4ème Série, Section Botanique 3, no. 2: 173–177.

[ece370784-bib-1012] Kostermans, A. J. G. H. 1982. “The Genus Hopea (Dipterocarpaceae) in Ceylon, Sri Lanka.” Ceylon Journal of Science, Biological Sciences 15: 41–49.

[ece370784-bib-1013] Kostermans, A. J. G. H. 1983. “The Genus Shorea (Dipterocarpaceae).” Botanishe Jahrbuecher für Systamatik Pflanzengeschichte und Pflanzengeographie 104: 183–192.

[ece370784-bib-1014] Kostermans, A. J. G. H. 1992. A Handbook of the Dipterocarpaceae of Sri Lanka, 244. Sri Lanka: Wildlife Heritage Trust of Sri Lanka.

[ece370784-bib-0050] Liu, H. , E. J. Edwards , R. P. Freckleton , and C. P. Osborne . 2012. “Phylogenetic Niche Conservatism in C4 Grasses.” Oecologia 170, no. 3: 835–845. 22569558 10.1007/s00442-012-2337-5

[ece370784-bib-1015] Londoño, A. C. , E. Alvarez , E. Forero , and C. M. Morton . 1995. “A New Genus and Species of Dipterocarpaceae from the Neotropics. I. Introduction, Taxonomy, Ecology, and Distribution.” Brittonia 47: 225–236.

[ece370784-bib-0051] Losos, J. B. 2011. “Seeing the Forest for the Trees: The Limitations of Phylogenies in Comparative Biology.” American Naturalist 177, no. 6: 709–727. 10.1086/660020 21597249

[ece370784-bib-1017] Maycock, C. R. , C. J. Kettle , E. Khoo , et al. 2012. “A Revised Conservation Assessment of Dipterocarps in Sabah.” Biotropica 44, no. 5: 649–657.

[ece370784-bib-0054] Meerts, P. , Q. Rougelot , and M. Sosef . 2017. “Revision of the Genus Monotes (Dipterocarpaceae) in DR Congo, With Implications for Angola and Its Distinction From Marquesia.” Phytotaxa 308, no. 2: 151–205.

[ece370784-bib-0055] Momose, K. , T. Yumoto , T. Nagamitsu , et al. 1998. “Pollination Biology in a Lowland Dipterocarp Forest in Sarawak, Malaysia. I. Characteristics of the Plant‐Pollinator Community in a Lowland Dipterocarp Forest.” American Journal of Botany 85, no. 10: 1477–1501. 21684899

[ece370784-bib-0056] Nandy, S. , S. Ghosh , and S. Singh . 2021. “Assessment of Sal ( *Shorea robusta* ) Forest Phenology and Its Response to Climatic Variables in India.” Environmental Monitoring and Assessment 193, no. 9: 616. 34476606 10.1007/s10661-021-09356-9

[ece370784-bib-1018] Orme, C. D. L. 2013. “Caper: Comparative Analyses of Phylogenetics and Evolution in R.” Methods in Ecology and Evolution 3: 145.

[ece370784-bib-0057] Pagel, M. 1999. “Inferring the Historical Patterns of Biological Evolution.” Nature 401, no. 6756: 877–884. 10553904 10.1038/44766

[ece370784-bib-0059] Palmiotto, P. A. , S. J. Davies , K. A. Vogt , M. S. Ashton , D. J. Vogt , and P. S. Ashton . 2004. “Soil‐Related Habitat Specialization in Dipterocarp Rain Forest Tree Species in Borneo.” Journal of Ecology 92, no. 4: 609–623.

[ece370784-bib-0060] Paoli, G. D. , L. M. Curran , and D. R. Zak . 2006. “Soil Nutrients and Beta Diversity in the Bornean Dipterocarpaceae: Evidence for Niche Partitioning by Tropical Rain Forest Trees.” Journal of Ecology 94, no. 1: 157–170.

[ece370784-bib-0061] Pavoine, S. , and M. B. Bonsall . 2011. “Measuring Biodiversity to Explain Community Assembly a Unified Approach.” Biological Reviews 86, no. 4: 792–812. 21155964 10.1111/j.1469-185X.2010.00171.x

[ece370784-bib-0062] Peay, K. G. , P. G. Kennedy , S. J. Davies , S. Tan , and T. D. Bruns . 2010. “Potential Link Between Plant and Fungal Distributions in a Dipterocarp Rainforest: Community and Phylogenetic Structure of Tropical Ectomycorrhizal Fungi Across a Plant and Soil Ecotone.” New Phytologist 185, no. 2: 529–542. 19878464 10.1111/j.1469-8137.2009.03075.x

[ece370784-bib-1002] Poore, D. , and J. Sayer . 1991. The Management of Tropical Moist Forest Lands: Ecological Guidelines. Vol. 2. Gland, Switzerland: IUCN.

[ece370784-bib-1021] Potts, M. D. , P. S. Ashton , L. S. Kaufman , and J. B. Plotkin . 2002. “Habitat Patterns in Tropical Rain Forests: A Comparison of 105 Plots in Northwest Borneo.” Ecology 83, no. 10: 2782–2797.

[ece370784-bib-0063] Purvis, A. , M. CarDillo , R. Grenyer , and B. Collen . 2005. “Correlates of Extinction Risk: Phylogeny, Biology, Threat and Scale.” In Phylogeny and Conservation, edited by A. Purvis , J. L. Gittleman , and T. Brooks , 295–316. Cambridge: Cambridge University Press (Conservation Biology).

[ece370784-bib-0064] Qian, H. , and Y. Jin . 2016. “An Updated Megaphylogeny of Plants, a Tool for Generating Plant Phylogenies and an Analysis of Phylogenetic Community Structure.” Journal of Plant Ecology 9, no. 2: 233–239.

[ece370784-bib-0065] Qian, H. , and J. Zhang . 2014. “Using an Updated Time‐Calibrated Family‐Level Phylogeny of Seed Plants to Test for Non‐random Patterns of Life Forms Across the Phylogeny.” Journal of Systematics and Evolution 52, no. 4: 423–430.

[ece370784-bib-0066] Redding, D. W. , and A. O. Mooers . 2006. “Incorporating Evolutionary Measures Into Conservation Prioritization.” Conservation Biology 20, no. 6: 1670–1678. 17181802 10.1111/j.1523-1739.2006.00555.x

[ece370784-bib-1006] Revell, L. J. , L. J. Harmon , and D. C. Collar . 2008. “Phylogenetic Signal, Evolutionary Process, and Rate.” Systematic Biology 57, no. 4: 591–601. 18709597 10.1080/10635150802302427

[ece370784-bib-1022] Rineau, F. , and J. Garbaye . 2009. “Does Forest Liming Impact the Enzymatic Profiles of Ectomycorrhizal Communities Through Specialized Fungal Symbionts?” Mycorrhiza 19, no. 7: 493–500. 19421790 10.1007/s00572-009-0249-y

[ece370784-bib-0067] Russo, S. E. , S. J. Davies , D. A. King , and S. Tan . 2005. “Soil‐Related Performance Variation and Distributions of Tree Species in a Bornean Rain Forest.” Journal of Ecology 93, no. 5: 879–889.

[ece370784-bib-0068] Sahoo, H. , A. Kumar , Y. Mishra , and R. Kumar . 2024. “Genetic Assessment and Variation Study Among Different Populations of *Shorea robusta* Gaertn. F. in Jharkhand, India.” Ecology Environment and Conservation 30: 422–426. 10.53550/EEC.2024.v30i04s.070.

[ece370784-bib-0069] Sakai, S. 2002. “General Flowering in Lowland Mixed Dipterocarp Forests of South‐East Asia.” Biological Journal of the Linnean Society 75, no. 2: 233–247.

[ece370784-bib-1020] Shamshuddin, J. , and C. I. Fauziah . 2010. Weathered Tropical Soils: The Ultisols and Oxisols, Universiti Putra Malaysia Press. http://agris.fao.org/agris‐search/search.do?recordID=MY2014000389 [Accessed February 26, 2019].

[ece370784-bib-0070] Sjöström, A. , and C. L. Gross . 2006. “Life‐History Characters and Phylogeny Are Correlated With Extinction Risk in the Australian Angiosperms.” Journal of Biogeography 33, no. 2: 271–290.

[ece370784-bib-0071] Slik, J. F. , V. Arroyo‐Rodríguez , S. I. Aiba , et al. 2015. “An Estimate of the Number of Tropical Tree Species.” Proceedings of the National Academy of Sciences 112, no. 24: 7472–7477. 10.1073/pnas.1423147112 PMC4475970 26034279

[ece370784-bib-1003] Swamy, V. , and J. W. Terborgh . 2010. “Distance‐Responsive Natural Enemies Strongly Influence Seedling Establishment Patterns of Multiple Species in an Amazonian Rain Forest.” Journal of Ecology 98, no. 5: 1096–1107.

[ece370784-bib-1008] Symington, C. F. 1974. Forester's Manual of Dipterocarps. Malaysian Forest Records No. 16 (First Published in 1943, Reprinted in 1974), Kuala Lumpur, Malaysia.

[ece370784-bib-0075] Tedersoo, L. , T. Suvi , K. Beaver , and U. Kõljalg . 2007. “Ectomycorrhizal Fungi of the Seychelles: Diversity Patterns and Host Shifts From the Native Vateriopsis Seychellarum (Dipterocarpaceae) and *Intsia bijuga* (Caesalpiniaceae) to the Introduced *Eucalyptus robusta* (Myrtaceae), but Not P.” New Phytologist 175: 321–333. 17587380 10.1111/j.1469-8137.2007.02104.x

[ece370784-bib-0076] Warren‐Thomas, E. , P. M. Dolman , and D. P. Edwards . 2015. “Increasing Demand for Natural Rubber Necessitates a Robust Sustainability Initiative to Mitigate Impacts on Tropical Biodiversity.” Conservation Letters 8, no. 4: 230–241.

[ece370784-bib-0078] Whitmore, T. C. 1984. “A Vegetation Map of Malesia at Scale 1:5 Million.” Journal of Biogeography 11, no. 6: 461–471.

[ece370784-bib-0079] Widiyatno, W. , F. Hidayati , S. Hardiwinoto , et al. 2020. “Selection of Dipterocarp Species for Enrichment Planting in a Secondary Tropical Rainforest.” Forest Science and Technology 16, no. 4: 206–215.

[ece370784-bib-0081] Wiens, J. J. , and C. H. Graham . 2005. “Niche Conservatism: Integrating Evolution, Ecology, and Conservation Biology.” Annual Review of Ecology, Evolution, and Systematics 36, no. 1: 519–539.

[ece370784-bib-0082] Wilcove, D. S. , X. Giam , D. P. Edwards , B. Fisher , and L. P. Koh . 2013. “Navjot's Nightmare Revisited: Logging, Agriculture, and Biodiversity in Southeast Asia.” Trends in Ecology & Evolution 28, no. 9: 531–540. 23764258 10.1016/j.tree.2013.04.005

[ece370784-bib-0083] Willis, C. G. , B. Ruhfel , R. B. Primack , A. J. Miller‐Rushing , and C. C. Davis . 2008. “Phylogenetic Patterns of Species Loss in Thoreau's Woods Are Driven by Climate Change.” Proceedings of the National Academy of Sciences 105, no. 44: 17029–17033. 10.1073/pnas.0806446105 PMC2573948 18955707

[ece370784-bib-1024] Yeong, K. L. , G. Reynolds , and J. K. Hill . 2016. “Enrichment Planting to Improve Habitat Quality and Conservation Value of Tropical Rainforest Fragments.” Biodiversity and Conservation 25: 957–973.

[ece370784-bib-0084] Zanne, A. E. , D. C. Tank , W. K. Cornwell , et al. 2014. “Three Keys to the Radiation of Angiosperms Into Freezing Environments.” Nature 506, no. 7486: 89–92. 24362564 10.1038/nature12872

[ece370784-bib-1005] Zhu, K. , C. W. Woodall , J. V. Monteiro , and J. S. Clark . 2015. “Prevalence and Strength of Density‐Dependent Tree Recruitment.” Ecology 96, no. 9: 2319–2327. 26594690 10.1890/14-1780.1

