# Supplementary material for: Comparative Analyses and Phylogenetic Dependence in Traits and Trends of the Dipterocarpaceae
Source: Ecol Evol. 2025 Jan 9;15(1):e70784. doi: 10.1002/ece3.70784 (PMC11717900; doi:10.1002/ece3.70784)
Supplement: Supplementary file 1 — Appendix S1. [file ECE3-15-e70784-s002.docx]

**Appendix C**: List of plant traits of dipterocarp species that have been used in this study

| Traits | Definition | Units | Description of classes |
| --- | --- | --- | --- |
| Lower elevation limit | Low distance above sea level of species occurrence | m | Quantitative value |
| Upper elevation limit | High distance above sea level of species occurrence | m | Quantitative value |
| Endemism | Species that being unique to specific location |  | Qualitative (Widespread = 0, Endemic= 1) |
| Estimated Extent of Occurrence | “area contained within the shortest continuous imaginary boundary which can be drawn to encompass all the known, inferred or projected sites of present occurrence of a taxon, excluding cases of vagrancy”, (IUCN 2001) | km^2^ | Quantitative value |
| Estimated Area of Occupancy | “area within its 'extent of occurrence' which is occupied by a taxon, excluding cases of vagrancy”, (IUCN 2001) | km^2^ | Quantitative value |
| Habitat Soil type | Soil type that inhabited by plant (Voroney, 2007) |  | Qualitative (Soil type= Clay, Sandy, Loam, and Limestone  Inhabiting? Yes= 1, No=0) |
| Height | Distance from ground level to the level top of the tree | m | Quantitative value |
| Diameter at breast height (DBH) | Measurement of tree stem at the height of 1.30 m | cm | Quantitative value |
| Growth rate | Girth increment per year | cm/per year | Quantitative value |
| Shade tolerance | Ability to tolerate low light level |  | Qualitative (Shade tolerant= 0, Intermediate= 1, Light demander= 2) |
| Leaf length | Length of the leaf in vascular plants from lamina tip to the petioles along lamina midrib (Cho *et al.*, 2007) | cm | Quantitative value |
| Flower size | Diameter of flower | mm | Qualitative (Small(<10mm) = 0, Medium(10—20mm) = 1, Large(>20mm) = 2) |
| Flower reward | Secretion or structure of the labellum that can be consumed or gathered by pollinators (Singer & Koehler 2004) |  | Qualitative (Type= Nectar, Pollen and Corolla, Produced? Yes= 1, No= 0) |
| Survival | Tree mortality | % | Quantitative value |
| Flowering frequency | Regularity of flowering in vascular plant |  | Qualitative (General= 0, Regular= 1) |
| Anthesis (Day) | Flowering period of plant (0600-1800) |  | Qualitative (Yes= 1, No= 0) |
| Anthesis (Night) | Flowering period of plant (1800-0600) |  | Qualitative (Yes=1, No=0) |
| Chromosome number | Number of DNA molecule that carry genetic information of plant (Battaglia, 1955) |  | Qualitative (Chromosome no x=7,10,11 and Polyploidy,  Yes=1, No=0) |
| Outcrossing rate | Rates of crossing between different breeds | % | Quantitative value |
| Fruit length | Length of nut | mm | Quantitative value |
| Fruit width | Width of nut | mm | Quantitative value |
| Seed weight | Seed mass | seed per kilo | Quantitative value |
| Functional wing | Wings that involved in seed dispersal |  | Qualitative (Wing no= 0,2,3 and 5,  Has? Yes= 1, No=0) |
| Functional wing length | Measurement of length of wings involved in dispersal | mm | Quantitative value |
| Wing loading | Fruit mass divided by wing surface area(Green, 1980) |  | Quantitative value |
| Wood type | Hardwood type classification |  | Qualitative (Light Hardwood=0, Medium Hardwood= 1, Heavy Hardwood= 2) |
| Wood densities | “Measurement of the ratio of oven-dry mass of wood divided mass of water displaced by its green volume “ (Chave, no date) | g/cm^3^ | Quantitative value |
| Red List status | Species conservation status through criteria such as population size, rate of decline and geographic distribution as listed in IUCN Red List Categories (IUCN 2017) |  | Qualitative (Data Deficient= 0, Least Concern= 1, Near Threatened= 2, Vulnerable= 3, Endangered=4, Critically Endangered=5, Extinct in The Wild= 6) |

**Appendix C1**: Dipterocarp species list used in the study

| *Anisoptera brunnea* | *Dipterocarpus coriaceus* |
| --- | --- |
| *Anisoptera costata* | *Dipterocarpus cornutus* |
| *Anisoptera curtisii* | *Dipterocarpus costatus* |
| *Anisoptera grossivenia* | *Dipterocarpus costulatus* |
| *Anisoptera laevis* | *Dipterocarpus crinitus* |
| *Anisoptera marginata* | *Dipterocarpus cuspidatus* |
| *Anisoptera megistocarpa* | *Dipterocarpus dyeri* |
| *Anisoptera reticulata* | *Dipterocarpus elongatus* |
| *Anisoptera scaphula* | *Dipterocarpus eurhynchus* |
| *Anisoptera thurifera* | *Dipterocarpus fagineus* |
| *Cotylelobium burckii* | *Dipterocarpus fusiformis* |
| *Cotylelobium lanceolatum* | *Dipterocarpus geniculatus* |
| *Cotylelobium lewisianum* | *Dipterocarpus glabrigemmatus* |
| *Cotylelobium melanoxylon* | *Dipterocarpus glandulosus* |
| *Cotylelobium scabriusculum* | *Dipterocarpus globosus* |
| *Dipterocarpus acutangulus* | *Dipterocarpus gonopterus* |
| *Dipterocarpus alatus* | *Dipterocarpus gracilis* |
| *Dipterocarpus applanatus* | *Dipterocarpus grandiflorus* |
| *Dipterocarpus baudii* | *Dipterocarpus hasseltii* |
| *Dipterocarpus borneensis* | *Dipterocarpus hispidus* |
| *Dipterocarpus bourdillonii* | *Dipterocarpus humeratus* |
| *Dipterocarpus caudatus* | *Dipterocarpus indicus* |
| *Dipterocarpus caudiferus* | *Dipterocarpus insignis* |
| *Dipterocarpus chartaceus* | *Dipterocarpus intricatus* |
| *Dipterocarpus cinereus* | *Dipterocarpus kerrii* |
| *Dipterocarpus concavus* | *Dipterocarpus kunstleri* |
| *Dipterocarpus confertus* | *Dipterocarpus lamellatus* |
| *Dipterocarpus conformis* | *Dipterocarpus littoralis* |
| *Dipterocarpus lowii* | *Dryobalanops fusca* |
| *Dipterocarpus mannii* | *Dryobalanops keithii* |
| *Dipterocarpus megacarpus* | *Dryobalanops lanceolata* |
| *Dipterocarpus mundus* | *Dryobalanops oblongifolia* |
| *Dipterocarpus nudus* | *Dryobalanops rappa* |
| *Dipterocarpus oblongifolius* | *Dryobalanops sumatrensis* |
| *Dipterocarpus obtusifolius* | *Hopea acuminata* |
| *Dipterocarpus ochraceus* | *Hopea aequalis* |
| *Dipterocarpus orbicularis* | *Hopea altocollina* |
| *Dipterocarpus pachyphyllus* | *Hopea andersonii* |
| *Dipterocarpus palembanicus* | *Hopea apiculata* |
| *Dipterocarpus perakensis* | *Hopea aptera* |
| *Dipterocarpus pseudocornutus* | *Hopea auriculata* |
| *Dipterocarpus retusus* | *Hopea bancana* |
| *Dipterocarpus rigidus* | *Hopea basilanica* |
| *Dipterocarpus rotundifolius* | *Hopea beccariana* |
| *Dipterocarpus sarawakensis* | *Hopea bilitonensis* |
| *Dipterocarpus semivestitus* | *Hopea brachyptera* |
| *Dipterocarpus stellatus* | *Hopea bracteata* |
| *Dipterocarpus sublamellatus* | *Hopea brevipetiolaris* |
| *Dipterocarpus tempehes* | *Hopea bullatifolia* |
| *Dipterocarpus tuberculatus* | *Hopea cagayanensis* |
| *Dipterocarpus turbinatus* | *Hopea canarensis* |
| *Dipterocarpus validus* | *Hopea celebica* |
| *Dipterocarpus verrucosus* | *Hopea celtidifolia* |
| *Dipterocarpus zeylanicus* | *Hopea centipeda* |
| *Dryobalanops aromatica* | *Hopea cernua* |
| *Dryobalanops beccarii* | *Hopea chinensis* |
| *Hopea cordata* | *Hopea jucunda* |
| *Hopea cordifolia* | *Hopea kerangasensis* |
| *Hopea coriacea* | *Hopea kitulgallensis* |
| *Hopea dasyrrhachis* | *Hopea latifolia* |
| *Hopea depressinerva* | *Hopea longirostrata* |
| *Hopea discolor* | *Hopea malibato* |
| *Hopea dryobalanoides* | *Hopea megacarpa* |
| *Hopea dyeri* | *Hopea mengarawan* |
| *Hopea enicosanthoides* | *Hopea mesuoides* |
| *Hopea erosa* | *Hopea micrantha* |
| *Hopea exalata* | *Hopea mindanensis* |
| *Hopea ferrea* | *Hopea modesta* |
| *Hopea ferruginea* | *Hopea mollissima* |
| *Hopea fluvialis* | *Hopea montana* |
| *Hopea forbesii* | *Hopea myrtifolia* |
| *Hopea foxworthyi* | *Hopea nervosa* |
| *Hopea glabra* | *Hopea nigra* |
| *Hopea glabrifolia* | *Hopea nodosa* |
| *Hopea glaucescens* | *Hopea novoguineensis* |
| *Hopea gregaria* | *Hopea nutans* |
| *Hopea griffithii* | *Hopea oblongifolia* |
| *Hopea hainanensis* | *Hopea obscurinerva* |
| *Hopea helferi* | *Hopea odorata* |
| *Hopea hongayanensis* | *Hopea ovoidea* |
| *Hopea inexpectata* | *Hopea pachycarpa* |
| *Hopea iriana* | *Hopea papuana* |
| *Hopea jacobi* | *Hopea parviflora* |
| *Hopea johorensis* | *Hopea parvifolia* |
| *Hopea paucinervis* | *Hopea treubii* |
| *Hopea pedicellata* | *Hopea ultima* |
| *Hopea pentanervia* | *Hopea utilis* |
| *Hopea philippinensis* | *Hopea vacciniifolia* |
| *Hopea pierrei* | *Hopea vesquei* |
| *Hopea plagata* | *Hopea vietnamensis* |
| *Hopea polyalthioides* | *Hopea wyatt-smithii* |
| *Hopea ponga* | *Marquesia acuminata* |
| *Hopea pterygota* | *Marquesia excelsa* |
| *Hopea pubescens* | *Marquesia macroura* |
| *Hopea quisumbingiana* | *Monotes adenophyllus* |
| *Hopea racophloea* | *Monotes africanus* |
| *Hopea recopei* | *Monotes autennei* |
| *Hopea reticulata* | *Monotes dasyanthus* |
| *Hopea rudiformis* | *Monotes doryphorus* |
| *Hopea rugifolia* | *Monotes duvigneaudii* |
| *Hopea samarensis* | *Monotes engleri* |
| *Hopea sangal* | *Monotes glaber* |
| *Hopea scabra* | *Monotes glandulosus* |
| *Hopea semicuneata* | *Monotes gossweileri* |
| *Hopea shingkeng* | *Monotes hirtii* |
| *Hopea similis* | *Monotes hypoleucus* |
| *Hopea sphaerocarpa* | *Monotes katangensis* |
| *Hopea subalata* | *Monotes kerstingii* |
| *Hopea sublanceolata* | *Monotes lutambensis* |
| *Hopea sulcata* | *Monotes madagascariensis* |
| *Hopea tenuinervula* | *Monotes magnificus* |
| *Hopea thorelii* | *Monotes paivae* |
| *Monotes pearsonii* | *Shorea almon* |
| *Monotes redheadii* | *Shorea altopoensis* |
| *Monotes rubriglans* | *Shorea alutacea* |
| *Monotes rufotomentosus* | *Shorea amplexicaulis* |
| *Monotes xasenguensis* | *Shorea andulensis* |
| *Neobalanocarpus heimii* | *Shorea angustifolia* |
| *Pakaraimaea dipterocarpacea* | *Shorea argentea* |
| *Parashorea aptera* | *Shorea argentifolia* |
| *Parashorea buchananii* | *Shorea asahii* |
| *Parashorea chinensis* | *Shorea assamica* |
| *Parashorea densiflora* | *Shorea astylosa* |
| *Parashorea dussaudii* | *Shorea atrinervosa* |
| *Parashorea globosa* | *Shorea bakoensis* |
| *Parashorea lucida* | *Shorea balangeran* |
| *Parashorea macrophylla* | *Shorea balanocarpoides* |
| *Parashorea malaanonan* | *Shorea beccariana* |
| *Parashorea parvifolia* | *Shorea bentongensis* |
| *Parashorea smythiesii* | *Shorea biawak* |
| *Parashorea stellata* | *Shorea blumutensis* |
| *Parashorea tomentella* | *Shorea bracteolata* |
| *Parashorea warburgii* | *Shorea brunnescens* |
| *Pseudomonotes tropenbosii* | *Shorea bullata* |
| *Shorea acuminata* | *Shorea calcicola* |
| *Shorea acuminatissima* | *Shorea cara* |
| *Shorea acuta* | *Shorea carapae* |
| *Shorea affinis* | *Shorea chaiana* |
| *Shorea agamii* | *Shorea ciliata* |
| *Shorea albida* | *Shorea collaris* |
| *Shorea collina* | *Shorea flemmichii* |
| *Shorea confusa* | *Shorea foraminifera* |
| *Shorea congestiflora* | *Shorea foxworthyi* |
| *Shorea conica* | *Shorea furfuracea* |
| *Shorea contorta* | *Shorea gardneri* |
| *Shorea cordata* | *Shorea geniculata* |
| *Shorea cordifolia* | *Shorea gibbosa* |
| *Shorea coriacea* | *Shorea glauca* |
| *Shorea crassa* | *Shorea gratissima* |
| *Shorea curtisii* | *Shorea guiso* |
| *Shorea cuspidata* | *Shorea havilandii* |
| *Shorea dasyphylla* | *Shorea hemsleyana* |
| *Shorea dealbata* | *Shorea henryana* |
| *Shorea dispar* | *Shorea hopeifolia* |
| *Shorea disticha* | *Shorea hulanidda* |
| *Shorea domatiosa* | *Shorea hypochra* |
| *Shorea dyeri* | *Shorea hypoleuca* |
| *Shorea elliptica* | *Shorea iliasii* |
| *Shorea exelliptica* | *Shorea inaequilateralis* |
| *Shorea faguetiana* | *Shorea inappendiculata* |
| *Shorea faguetioides* | *Shorea induplicata* |
| *Shorea falcata* | *Shorea isoptera* |
| *Shorea falcifera* | *Shorea javanica* |
| *Shorea falciferoides* | *Shorea johorensis* |
| *Shorea fallax* | *Shorea kuantanensis* |
| *Shorea farinosa* | *Shorea kudatensis* |
| *Shorea ferruginea* | *Shorea kunstleri* |
| *Shorea flaviflora* | *Shorea ladiana* |
| *Shorea laevis* | *Shorea oblongifolia* |
| *Shorea lamellata* | *Shorea obovoidea* |
| *Shorea laxa* | *Shorea obscura* |
| *Shorea lepidota* | *Shorea obtusa* |
| *Shorea leprosula* | *Shorea ochracea* |
| *Shorea leptoderma* | *Shorea ochrophloia* |
| *Shorea lissophylla* | *Shorea ovalifolia* |
| *Shorea longiflora* | *Shorea ovalis* |
| *Shorea longisperma* | *Shorea ovata* |
| *Shorea lumutensis* | *Shorea pachyphylla* |
| *Shorea lunduensis* | *Shorea palembanica* |
| *Shorea macrantha* | *Shorea pallescens* |
| *Shorea macrobalanos* | *Shorea pallidifolia* |
| *Shorea macrophylla* | *Shorea palosapis* |
| *Shorea macroptera* | *Shorea parvifolia* |
| *Shorea malibato* | *Shorea parvistipulata* |
| *Shorea materialis* | *Shorea patoiensis* |
| *Shorea maxima* | *Shorea pauciflora* |
| *Shorea maxwelliana* | *Shorea peltata* |
| *Shorea mecistopteryx* | *Shorea pilosa* |
| *Shorea megistophylla* | *Shorea pinanga* |
| *Shorea micans* | *Shorea platycarpa* |
| *Shorea monticola* | *Shorea platyclados* |
| *Shorea montigena* | *Shorea polita* |
| *Shorea mujongensis* | *Shorea polyandra* |
| *Shorea multiflora* | *Shorea polysperma* |
| *Shorea myrionerva* | *Shorea praestans* |
| *Shorea negrosensis* | *Shorea pubistyla* |
| *Shorea quadrinervis* | *Shorea submontana* |
| *Shorea resinosa* | *Shorea sumatrana* |
| *Shorea retinodes* | *Shorea superba* |
| *Shorea retusa* | *Shorea symingtonii* |
| *Shorea revoluta* | *Shorea tenuiramulosa* |
| *Shorea richetia* | *Shorea teysmanniana* |
| *Shorea robusta* | *Shorea thorelii* |
| *Shorea rogersiana* | *Shorea trapezifolia* |
| *Shorea rotundifolia* | *Shorea tumbuggaia* |
| *Shorea roxburghii* | *Shorea uliginosa* |
| *Shorea rubella* | *Shorea venulosa* |
| *Shorea rubra* | *Shorea virescens* |
| *Shorea rugosa* | *Shorea waltoni* |
| *Shorea sagittata* | *Shorea wangtianshuea* |
| *Shorea scaberrima* | *Shorea woodii* |
| *Shorea scabrida* | *Shorea worthingtoni* |
| *Shorea scrobiculata* | *Shorea xanthophylla* |
| *Shorea selanica* | *Shorea zeylanica* |
| *Shorea seminis* | *Stemonoporus acuminatus* |
| *Shorea siamensis* | *Stemonoporus affinis* |
| *Shorea singkawang* | *Stemonoporus angustisepalus* |
| *Shorea slootenii* | *Stemonoporus bullatus* |
| *Shorea smithiana* | *Stemonoporus canaliculatus* |
| *Shorea splendida* | *Stemonoporus cordifolius* |
| *Shorea squamata* | *Stemonoporus elegans* |
| *Shorea stenoptera* | *Stemonoporus gardneri* |
| *Shorea stipularis* | *Stemonoporus gilimalensis* |
| *Shorea subcylindrica* | *Stemonoporus gracilis* |
| *Stemonoporus kanneliyensis* | *Vatica borneensis* |
| *Stemonoporus laevifolius* | *Vatica brevipes* |
| *Stemonoporus lanceolatus* | *Vatica brunigii* |
| *Stemonoporus lancifolius* | *Vatica cauliflora* |
| *Stemonoporus latisepalus* | *Vatica chartacea* |
| *Stemonoporus marginalis* | *Vatica chevalieri* |
| *Stemonoporus mooni* | *Vatica chinensis* |
| *Stemonoporus nitidus* | *Vatica cinerea* |
| *Stemonoporus oblongifolius* | *Vatica compressa* |
| *Stemonoporus petiolaris* | *Vatica congesta* |
| *Stemonoporus reticulatus* | *Vatica coriacea* |
| *Stemonoporus revolutus* | *Vatica cuspidata* |
| *Stemonoporus rigidus* | *Vatica diospyroides* |
| *Stemonoporus scalarinervis* | *Vatica dulitensis* |
| *Stemonoporus scaphifolius* | *Vatica elliptica* |
| *Stemonoporus zeylanicus* | *Vatica endertii* |
| *Upuna borneensis* | *Vatica flavida* |
| *Vateria copallifera* | *Vatica flavovirens* |
| *Vateria indica* | *Vatica glabrata* |
| *Vateria macrocarpa* | *Vatica globosa* |
| *Vateriopsis seychellarum* | *Vatica granulata* |
| *Vatica abdulrahmaniana* | *Vatica griffithii* |
| *Vatica adenanii* | *Vatica guangxiensis* |
| *Vatica affinis* | *Vatica harmandiana* |
| *Vatica albiramis* | *Vatica havilandii* |
| *Vatica badiifolia* | *Vatica heteroptera* |
| *Vatica bantamensis* | *Vatica hullettii* |
| *Vatica bella* | *Vatica javanica* |
| *Vatica lanceifolia* | *Vatica sarawakensis* |
| *Vatica lobata* | *Vatica scortechinii* |
| *Vatica lowii* | *Vatica soepadmoi* |
| *Vatica maingayi* | *Vatica stapfiana* |
| *Vatica mangachapoi* | *Vatica subglabra* |
| *Vatica maritima* | *Vatica teysmanniana* |
| *Vatica micrantha* | *Vatica thorelii* |
| *Vatica mizaniana* | *Vatica umbonata* |
| *Vatica nitens* | *Vatica venulosa* |
| *Vatica oblongifolia* | *Vatica vinosa* |
| *Vatica obovata* | *Vatica xishuangbannaensis* |
| *Vatica obscura* | *Vatica yeechongii* |
| *Vatica odorata* |  |
| *Vatica pachyphylla* |  |
| *Vatica pallida* |  |
| *Vatica paludosa* |  |
| *Vatica palungensis* |  |
| *Vatica parvifolia* |  |
| *Vatica patentinervia* |  |
| *Vatica pauciflora* |  |
| *Vatica pedicellata* |  |
| *Vatica pentandra* |  |
| *Vatica perakensis* |  |
| *Vatica philastreana* |  |
| *Vatica rassak* |  |
| *Vatica ridleyana* |  |
| *Vatica rotata* |  |
| *Vatica rynchocarpa* |  |

**Appendix C2**: Dipterocarp phylogeny in three scenarios. A) Scenario One; B) Scenario Two; C) Scenario Three


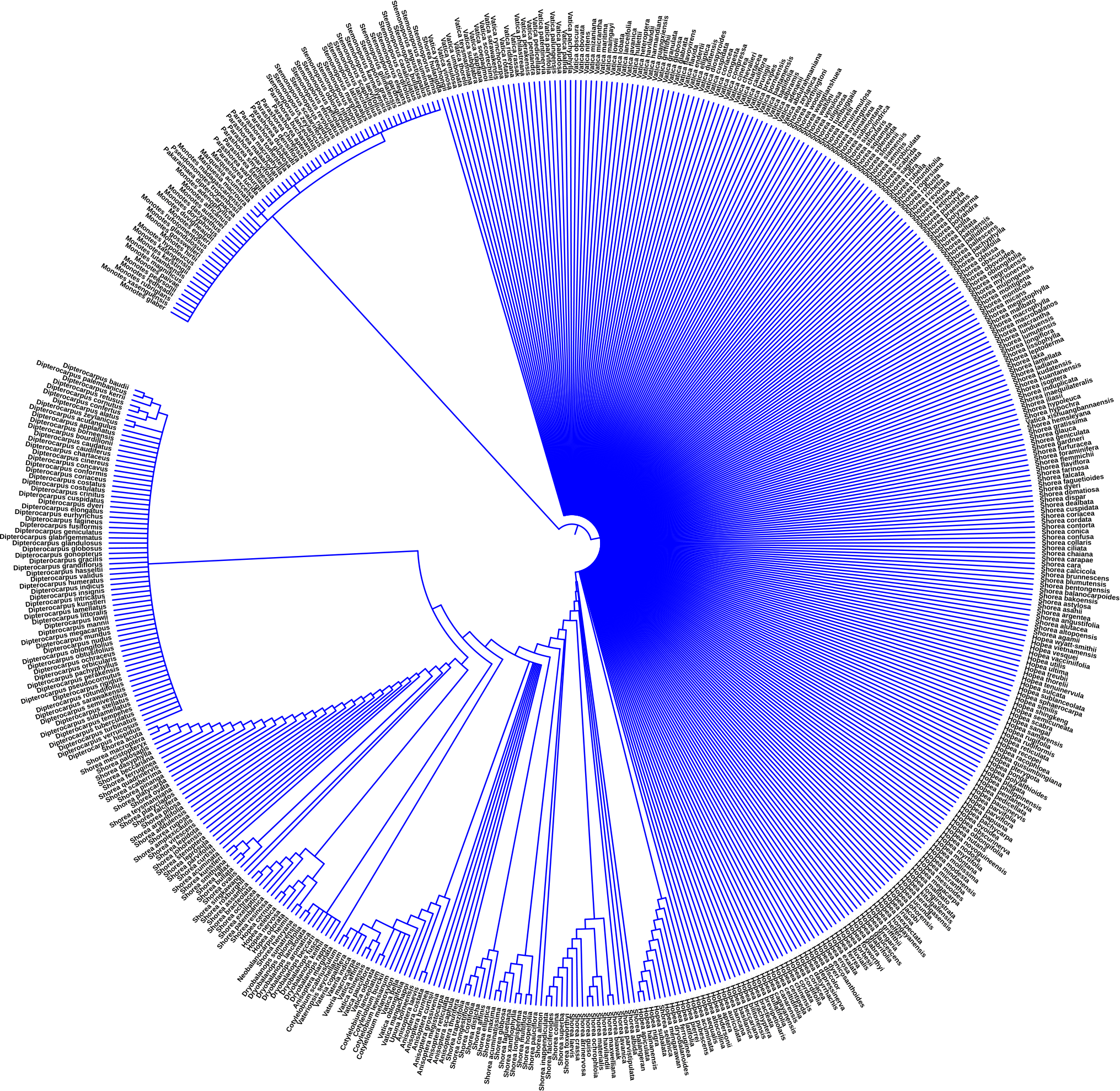


**A) Scenario One**


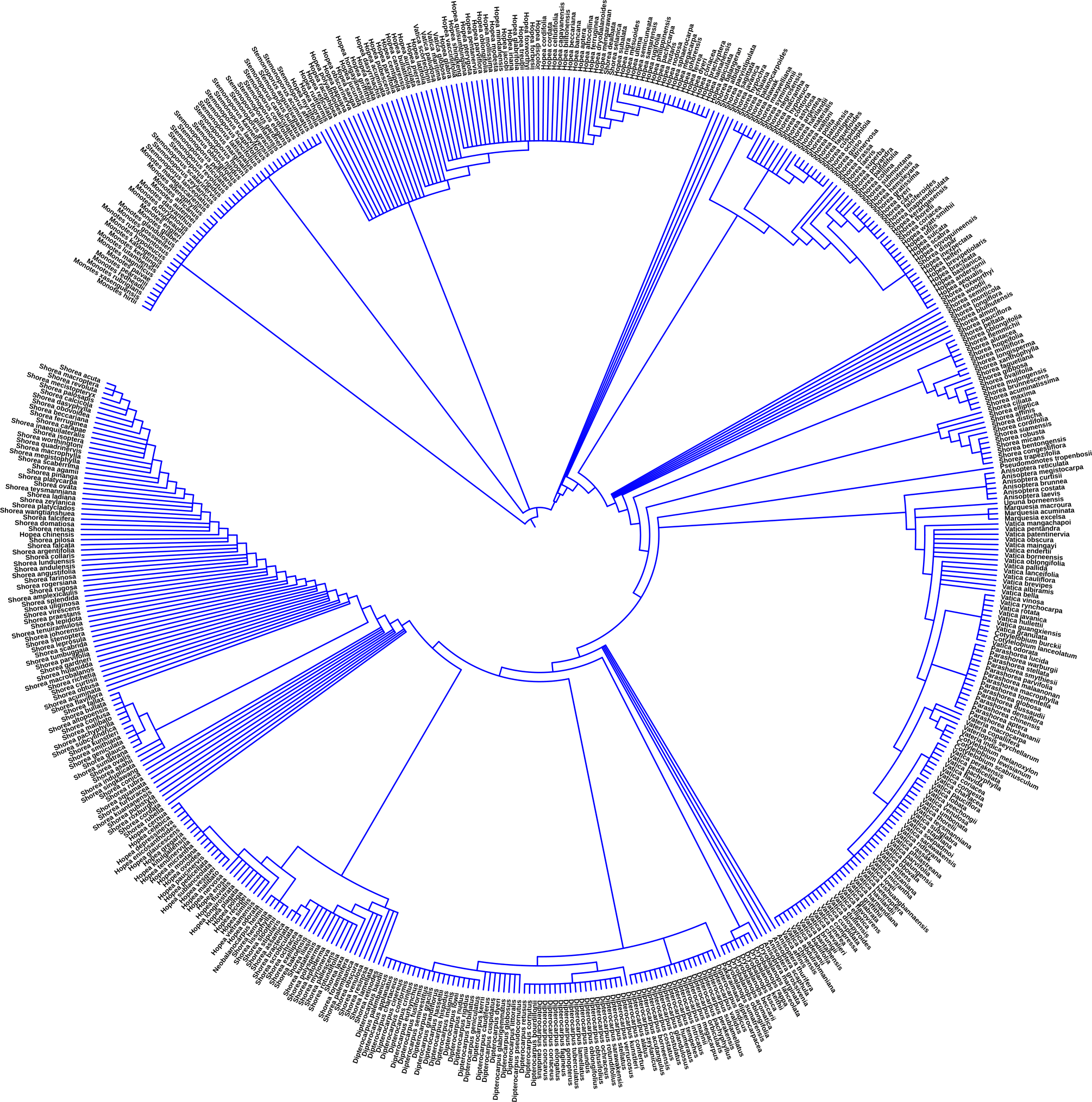


**B) Scenario Two**


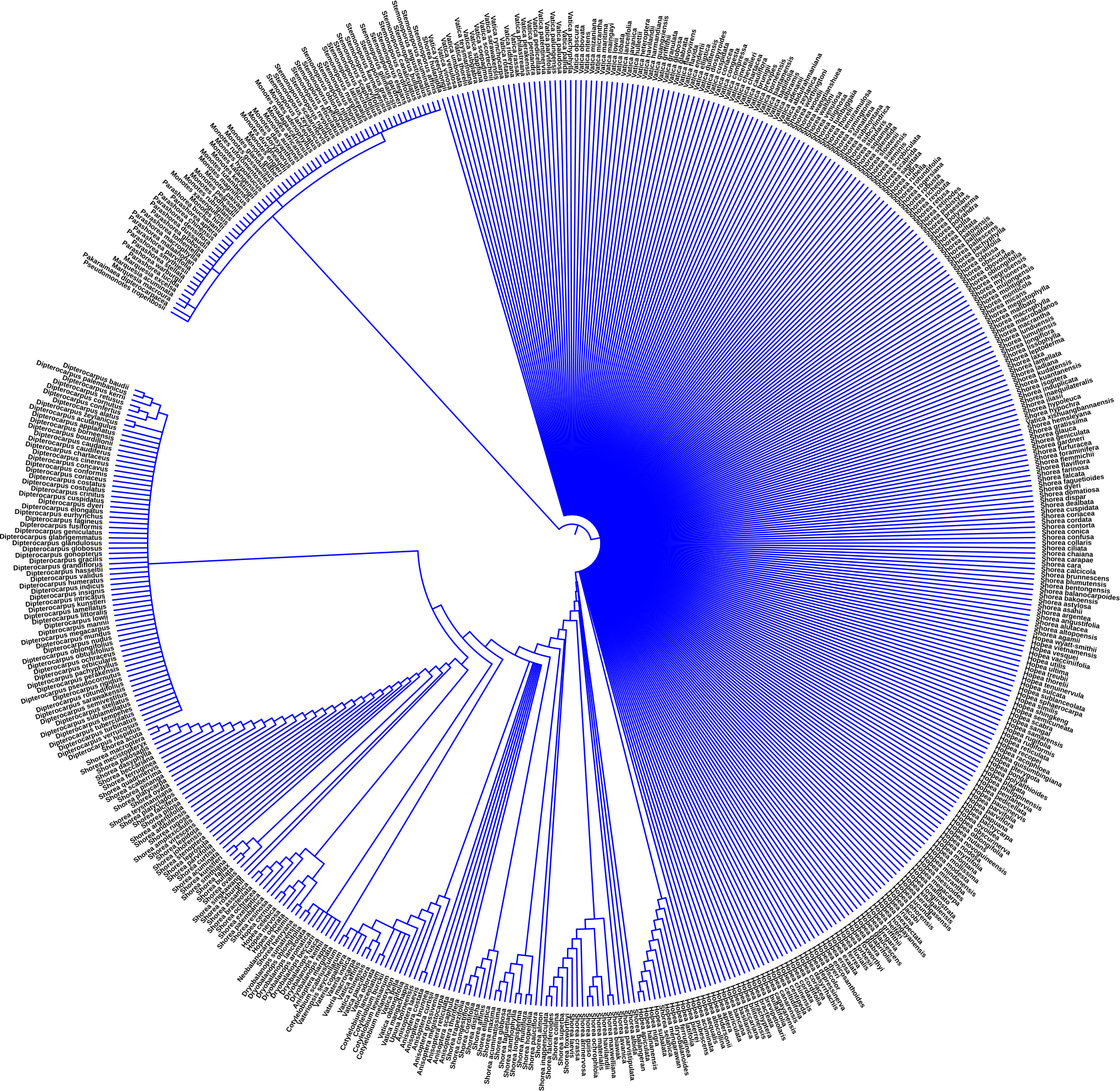


**C) Scenario Three**
