# Supplementary material for: Comparative Analyses and Phylogenetic Dependence in Traits and Trends of the Dipterocarpaceae
Source: Ecol Evol. 2025 Jan 9;15(1):e70784. doi: 10.1002/ece3.70784 (PMC11717900; doi:10.1002/ece3.70784)
Supplement: Supplementary file 4 — Table S1. [file ECE3-15-e70784-s003.docx]

**Table S1**: List of plant traits of dipterocarp species that have been used in this study

| Traits | Definition | Units | Description of classes |
| --- | --- | --- | --- |
| Lower elevation limit | Low distance above sea level of species occurrence | m | Quantitative value |
| Upper elevation limit | High distance above sea level of species occurrence | m | Quantitative value |
| Endemism | Species that are unique to specific location |  | Qualitative (Widespread = 0, Endemic= 1) |
| Estimated Extent of Occurrence | “Area contained within the shortest continuous imaginary boundary which can be drawn to encompass all the known, inferred or projected sites of present occurrence of a taxon, excluding cases of vagrancy”, (IUCN 2001) | km^2^ | Quantitative value |
| Estimated Area of Occupancy | “area within its 'extent of occurrence' which is occupied by a taxon, excluding cases of vagrancy”, (IUCN 2001) | km^2^ | Quantitative value |
| Habitat Soil type | Soil type that inhabited by plant (Voroney, 2007) |  | Qualitative (Soil type= Clay, Sandy, Loam, and Limestone  Inhabiting? Yes= 1, No=0) |
| Height | Distance from ground level to the level top of the tree | m | Quantitative value |
| Diameter at breast height (DBH) | Measurement of tree stem at the height of 1.30 m | cm | Quantitative value |
| Growth rate | Girth increment per year after planting | cm/per year | Quantitative value |
| Shade tolerance | Ability to tolerate low light level |  | Qualitative (Shade tolerant= 0, Intermediate= 1, Light demander= 2) |
| Leaf length | Length of the leaf in vascular plants from lamina tip to the petioles along lamina midrib (Cho *et al.*, 2007) | cm | Quantitative value |
| Flower size | Diameter of flower | mm | Qualitative (Small(<10mm) = 0, Medium(10—20mm) = 1, Large(>20mm) = 2) |
| Flower reward | Secretion or structure of the labellum that can be consumed or gathered by pollinators (Singer & Koehler 2004) |  | Qualitative (Type= Nectar, Pollen and Corolla, Produced? Yes= 1, No= 0) |
| Survival | Tree mortality | % | Quantitative value |
| Flowering frequency | Regularity of flowering in vascular plant |  | Qualitative (General= 0, Regular= 1) |
| Anthesis (Day) | Flowering period of plant (0600-1800) |  | Qualitative (Yes= 1, No= 0) |
| Anthesis (Night) | Flowering period of plant (1800-0600) |  | Qualitative (Yes=1, No=0) |
| Chromosome number | Number of DNA molecule that carry genetic information of plant (Battaglia, 1955) |  | Qualitative (Chromosome no x=7,10,11 and Polyploidy,  Yes=1, No=0) |
| Outcrossing rate | Rates of crossing between different breeds | % | Quantitative value |
| Fruit length | Length of nut | mm | Quantitative value |
| Fruit width | Width of nut | mm | Quantitative value |
| Seed weight | Seed mass | seed per kilo | Quantitative value |
| Functional wing | Wings that involved in seed dispersal |  | Qualitative (Wing no= 0,2,3 and 5,  Has? Yes= 1, No=0) |
| Functional wing length | Measurement of length of wings involved in dispersal | mm | Quantitative value |
| Wing loading | Fruit mass divided by wing surface area(Green, 1980) | cm^2^/g | Quantitative value |
| Wood type | Hardwood type classification |  | Qualitative (Light Hardwood=0, Medium Hardwood= 1, Heavy Hardwood= 2) |
| Wood densities | “Measurement of the ratio of oven-dry mass of wood divided mass of water displaced by its green volume “ (Chave, no date) | g/cm^3^ | Quantitative value |
| Red List status | Species conservation status through criteria such as population size, rate of decline and geographic distribution as listed in IUCN Red List Categories (IUCN 2017) |  | Qualitative (Data Deficient= 0, Least Concern= 1, Near Threatened= 2, Vulnerable= 3, Endangered=4, Critically Endangered=5, Extinct in The Wild= 6) |
| Habitat destruction | Alteration or elimination process by which a natural habitat becomes incapable of supporting its native species. |  | Qualitative (Yes=1, No=0) |
| Percentage of Habitat declined | Amount of habitat area declined due to deforestation and conversion into other land use. |  | Qualitative (Unknown percentage =0, Less than 30% = 1, Less than 50% = 2, Less than 80% = 3, More than 80% = 4) |
